# The demand for pregnancy testing: The Aschheim–Zondek reaction, diagnostic versatility, and laboratory services in 1930s Britain

**DOI:** 10.1016/j.shpsc.2013.12.002

**Published:** 2014-09

**Authors:** Jesse Olszynko-Gryn

**Affiliations:** Department of History and Philosophy of Science, University of Cambridge, Free School Lane, Cambridge CB2 3RH, UK

**Keywords:** Aschheim–Zondek test, Pregnancy hormone, Reproductive endocrinology, Diagnostic laboratory, Clinical pathology, Edinburgh

## Abstract

The Aschheim–Zondek reaction is generally regarded as the first reliable hormone test for pregnancy and as a major product of the ‘heroic age’ of reproductive endocrinology. Invented in Berlin in the late 1920s, by the mid 1930s a diagnostic laboratory in Edinburgh was performing thousands of tests every year for doctors around Britain. In her classic history of antenatal care, sociologist Ann Oakley claimed that the Aschheim–Zondek test launched a ‘modern era’ of obstetric knowledge, which asserted its superiority over that of pregnant women. This article reconsiders Oakley’s claim by examining how pregnancy testing worked in practice. It explains the British adoption of the test in terms less of the medicalisation of pregnancy than of clinicians’ increasing general reliance on laboratory services for differential diagnosis. Crucially, the Aschheim–Zondek reaction was a test not directly for the fetus, but for placental tissue. It was used, less as a yes-or-no test for ordinary pregnancy, than as a versatile diagnostic tool for the early detection of malignant tumours and hormonal deficiencies believed to cause miscarriage. This test was as much a product of oncology and the little-explored world of laboratory services as of reproductive medicine.

## Highlights

• Reconsiders pregnancy diagnosis alongside other laboratory services.
• Shows how diagnostic versatility was made into a major selling point of the Aschheim-Zondek test.
• Explains demand in terms of medical entrepreneurs and diagnostic consumers.

## 1 From innovation to routine

The Aschheim–Zondek reaction is generally regarded as the first modern test for the pregnancy hormone, today known as ‘human chorionic gonadotrophin’ or hCG. Though not the first laboratory pregnancy test, it was the first to be used on a large scale.^1^ Invented by Selmar Aschheim and Bernhard Zondek in Berlin in the late 1920s (Bröer, 2004; Finkelstein & Zondek, 1966; Hinz, Ebert, & Goetze, 1994; Rudloff & Ludwig, 2005; Schneck, 1997), by the mid 1930s a diagnostic service in Edinburgh was performing thousands of tests every year for clinicians and hospitals around Britain (Clarke, 1998, p. 320; Gurdon & Hopwood, 2000, pp. 45–46; Hanson, 2004, p. 136; McLaren, 2012, pp. 100–101; Oakley, 1984, p. 97; Wilmot, 2007, p. 433). Mice and rabbits, the story continues, were eventually replaced by the more efficient toad, *Xenopus laevis*, which in turn was supplanted by laboratory immunoassays and finally, in the early 1970s, by home test kits.^2^ Histories and ethnographies of reproduction have provided detailed analyses of newer and controversial diagnostic technologies including ultrasound, amniocentesis and genetic screening (Franklin & Roberts, 2006; Nicolson & Fleming, 2013; Rapp, 1999; Rothman, 1986), but pregnancy testing is often overlooked. In her classic history of antenatal care, sociologist Ann Oakley claimed that ‘the A-Z test launched the modern era in which obstetricians would eventually be able to claim a knowledge superior to that possessed by the owners of wombs themselves, as to the presence of a guest, invited or uninvited, within’ (Oakley, 1984, p. 98). Yet beyond the fact that the test was invented in Berlin and implemented on a large scale in Edinburgh, surprisingly little is known about how it worked in practice or the purposes for which it was used.

Above all, there is the problem of demand. Many women were aware of their menstrual cycles and familiar with the early signs of pregnancy, especially if they had already borne children (Usborne, 2007, p. 180). In early twentieth-century Britain, they rarely called on doctors or attended antenatal clinics before the second or third trimester, so it was unusual for medical practitioners to be involved in the early stages of pregnancy (Brookes, 1988, pp. 62–63). A woman who did seek out medical advice to confirm or allay her suspicions was usually told to return in a month’s time, unless ‘there was some particular reason why [she] should know’, in which case an Aschheim–Zondek test might be arranged (Oakley, 1984, pp. 97–98). Women who were contemplating abortion probably ‘preferred not to involve their GP in tests’ (Jones, 2007, p. 135). Rather, it was commonplace for women to take steps to bring on menstruation every month, a practice they did not equate with aborting a fetus (Fisher, 1999, pp. 221–222; Jones, 2007, p. 134). So if neither women nor doctors relied on the laboratory to help detect pregnancy, what was the Aschheim–Zondek test used for?

In this article I explain the adoption and institutionalisation of the Aschheim–Zondek test, in terms not of the medicalisation of ordinary pregnancy, but of clinicians’ increasing reliance on laboratory services for differential diagnosis. Crucially, the test ‘did not actually detect the presence of a live fetus’, but rather living placental tissue and so was ‘strongly positive’ for pathological growths such as hydatidiform mole or placental cancer, ‘where there was no viable fetus but plenty of chorionic epithelium.’^3^ Conversely, a weakly positive reaction could ‘indicate danger of miscarriage’ (McLaren, 2012, p. 101). I will show how the Aschheim–Zondek test was made, less into a yes-or-no test for normal pregnancy, and more into a versatile tool for differential diagnosis, calibrated to monitor placental tumours and hormonal deficiencies believed to cause miscarriage.^4^ I do not doubt that pregnancy tests were, as Adele Clarke put it, ‘early and important technoscientific products of the reproductive sciences’ (Clarke, 1998, p. 149), but innovation is not the whole story. A case study in use-based history of medical technology, my account will focus less on the novelties of scientific research than on the establishment and maintenance of routine practices.^5^ It will also situate pregnancy testing within the little-studied world of commercial laboratory services (Chen, 1992; Close-Koening, 2011; Crenner, 2006; Rosenberg, 1990; Worboys, 2004).

As Robert Kohler has recently observed, ‘laboratory history is now surprisingly neglected’ (Kohler, 2008, p. 761). Steve Sturdy and Roger Cooter’s account of statist efforts to rationalise health care remains an influential explanation of the rise of the laboratory in modern medicine (Kohler, 2008; Sturdy & Cooter, 1998). Their analysis is particularly good at explaining the role of the diagnostic laboratory in public health campaigns, for example, in mass screening programmes for syphilis or cervical cancer.^6^ But they missed an important piece of the puzzle: the medical market for commercial diagnostic testing, which was well established by the 1920s (Worboys, 2004). Laboratories sink or swim depending on ‘how effectively they [deal] with the rest of the world’, so it is important to look outside the laboratory and, in the case of diagnostic testing, beyond the managerial state as well, for the crucial ‘debates about what a laboratory should be, whether it is needed, by whom, and for what purposes—and about how it should be funded’ (Gooday, 2008, p. 786). As I will argue, the success or failure of pregnancy testing hinged on whether entrepreneurial testers managed to cultivate a viable commercial market beyond the lab and with minimal state support.^7^

An inviting model for historicising the diagnostic laboratory is Ludwik Fleck’s belated classic, *Genesis and development of a scientific fact*, first published in German in 1935. Fleck’s titular ‘fact’, which provided the empirical material for his general sociology of knowledge, was the relation between the Wassermann reaction and syphilis (Fleck, 1979). Fleck argued that the reaction became a clinically useful test many years after the initial ‘discovery’ paper of 1906. Rather than attempt to identify a single discoverer or turning point, Fleck instead emphasised the tedious labour of many anonymous laboratory workers in the ‘drawn-out process starting from false assumptions and irreproducible initial experiments along several dead ends and detours’ that made Wassermann’s reaction into a practical and reliable diagnostic tool (van den Belt, 2011, p. 332). At a more general level Fleck turned from what he perceived as the unreliably idealised and rationalised accounts of historical actors and eyewitnesses, including August von Wassermann, to a social view of collective discovery or invention. Though Fleck only mentioned the Aschheim–Zondek test in passing (to distance laboratory diagnosis from medieval uroscopy), in this article I want to take up his central sociological concerns with the significance of routine laboratory work and the sustained process of collective invention in the making of modern medicine and, in this case, modern pregnancy.^8^

## 2 Testing the test

An expectant mother who visited the antenatal clinic or was seen at home by a midwife in the early twentieth century might have had her blood pressure taken, her urine examined for albumin or sugar, or her blood tested for syphilis (O’Dowd & Philipp, 2000, p. 21), but it was not routine to test the urine of an apparently healthy woman to confirm pregnancy. By 1914, nearly half the adult population of Britain was covered by the 1911 National Health Insurance Act. Most women, all children, the elderly and self-employed were, however, excluded and benefits to women workers were cut in 1915 and again in 1932 (Digby & Bosanquet, 1988; Hardy, 2001, p. 80). Because they were unlikely to be covered by health insurance, working-class women did not usually visit a doctor except in an emergency (Brookes, 1988, p. 62). The act made no provision for laboratory services, so patients who could afford them paid out of pocket for diagnostic tests. Basic urinalysis was a side-room practice performed by a general practitioner, nurse, or midwife, but bacteriological and biochemical tests were left to clinical pathologists (Cunningham, 1992; Foster, 1961, 1983; Prüll, 1998, 2003). The wartime campaign against syphilis created state demand for mass Wassermann testing and the introduction of insulin and liver treatments in the 1920s increased interest in biochemical and haematological testing (Stevens, 1966). Routine analysis became increasingly structured around new divisions of labour and new specialities such as radiologists and pathologists who provided diagnostic services, not directly to patients, but to doctors (Amsterdamska & Hiddinga, 2003).

**Fig. 1:**
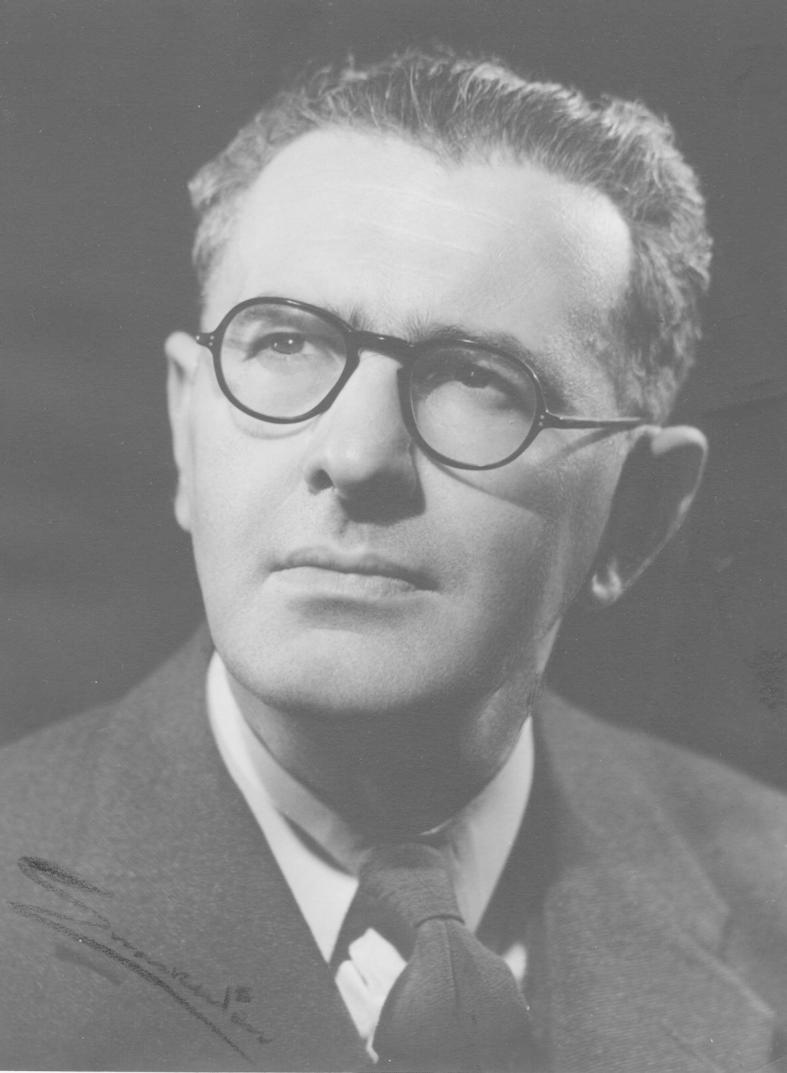
A previously unpublished photograph by Shackleton, Piccadilly, of Bertold Wiesner as a visionary scientist, undated but probably 1930s. Reproduced by kind permission of Jonathan Wiesner.

The Aschheim–Zondek reaction was first established in Britain at Francis Crew’s Department of Animal Breeding Research (later the Institute of Animal Genetics) at the University of Edinburgh (Clarke, 2007; Deacon, 1979; Hutt, 1931; Marie, 2004). Of the three animal breeding research institutes in 1920s Britain (at Cambridge, Edinburgh and Reading universities), this was the only one to branch out into medical research (Wilmot, 2007, p. 433). Although Crew was better known for his work on sex reversal and intersexuality in the domestic fowl, he also aspired to make a name for himself as an expert in human heredity, eugenics and social biology (Ha, 2011; Hogben, 1974; Porter, 1997; Richmond, 2007). But first he needed to medicalise his department, which was beholden to the Ministry of Agriculture. With help from Edinburgh professor of physiology Sir Edward Sharpey-Schafer, Crew attracted public and private donors for medical research, including controversial work on chemical spermicides (Borell, 1987; Löwy, 2009, 2011; Soloway, 1995). When Thomas B. Macaulay, a wealthy Canadian financier with Scottish ties, paid for a lectureship in endocrinology, Crew hired Bertold P. Wiesner, a young Austrian physiologist and ‘rejuvenationist’ he had met in 1926 at a Berlin Congress for Sex Research (Fig. 1).^9^

A product of Eugen Steinach’s controversial Institute of Experimental Biology in Vienna (the ‘Vivarium’), Wiesner modelled the ‘Macaulay Laboratory’ on that institution.^10^ When the Medical Research Council (MRC) refused Crew’s request for funding on the grounds that his institute was too agricultural, Crew turned to Robert W. Johnstone, the influential chair of the midwifery department, for support.^11^ Swayed by Johnstone, the MRC agreed to finance Wiesner’s work for one year.^12^ Wiesner and Crew began to collaborate with Johnstone, exchanging valuable research material (pregnant women’s urine and placentas) and access to patients for experimental therapeutic products (made from the urine and placentas) and access to laboratory animals.^13^

During the endocrine ‘gold rush’ of the 1920s and 1930s, drug companies isolated and mass-produced the internal secretions of the ovaries, testicles, pituitary and placenta (Borell, 1985; Gaudillière, 2005; Oudshoorn, 1994; Parkes, 1966). The Aschheim–Zondek reaction was a by-product of this ‘heroic age’ of reproductive endocrinology, or ‘sex physiology’ as it was then called, and Wiesner used it, not as a test for pregnancy, but to verify the potency of potentially therapeutic substances.^14^ Impressed by its efficacy in drug standardisation, he proposed to offer diagnostic testing as a routine service for doctors, beginning with Johnstone. He had three main reasons. First, the station would test the test on a large number of clinically unselected patients, thereby demonstrating the value of the agricultural institute to medical practitioners and researchers. Second, any surplus (hormonally rich) pregnancy urine sent to the station could be redirected towards research (injected into rats). Third, the station would charge a fee and so was expected to be self-financing or even to turn a profit that could be ploughed back into research, an economic strategy that other university and hospital laboratories were then adopting.^15^

Collaborating with Wiesner offered Johnstone several clear advantages too. First, with sex hormones a novelty in gynaecology, Wiesner supplied Johnstone with new and experimental therapeutic substances. The chance to test the expensive extracts on his private patients placed Johnstone at the forefront of clinical research. He also gained access to a new and potentially powerful diagnostic tool that could be tested on his hospital (and private) patients. A controversial specialist in infertility treatment, Johnstone used the Aschheim–Zondek test, not simply for pregnancy diagnosis, but to calibrate hormone injections in cases of endocrine deficiency believed to cause miscarriage.^16^ Last but not least, Johnstone needed Wiesner for animal injections, which were forbidden on infirmary property (Lawrence, 2005, p. 36; Sharpey-Schafer, 1930, p. 31).

Animal experiments, including routine injections, were permitted only in labs registered by the Home Office under the 1876 Cruelty to Animals Act and regularly spot-checked by medical inspectors. Every year, hundreds of thousands of animal injections were performed by the MRC, public health authorities, and private companies (under the Therapeutic Substances Act of 1925) in the routine production, testing, and standardisation of millions of doses of drugs, sera, and vaccines.^17^ These accounted for 95% of all licensed animal experiments in Britain and required ‘Certificate A’ (in addition to the license) to forego the use of anesthetics in mice and other species. As antivivisectionists gained public support in the late 1920s, hospital administrators became increasingly wary of losing the voluntary contributions of wealthy patrons and tended to keep animals away from hospital property (Tansey, 1994). For instance, the Middlesex Hospital in London used the animals kept at the Courtauld Institute of Biochemistry next door and the Royal Infirmary of Edinburgh fostered a cooperative attitude towards off-site laboratories (Lawrence, 2005, p. 66; Stone, 1932, p. 383).

**Fig. 2:**
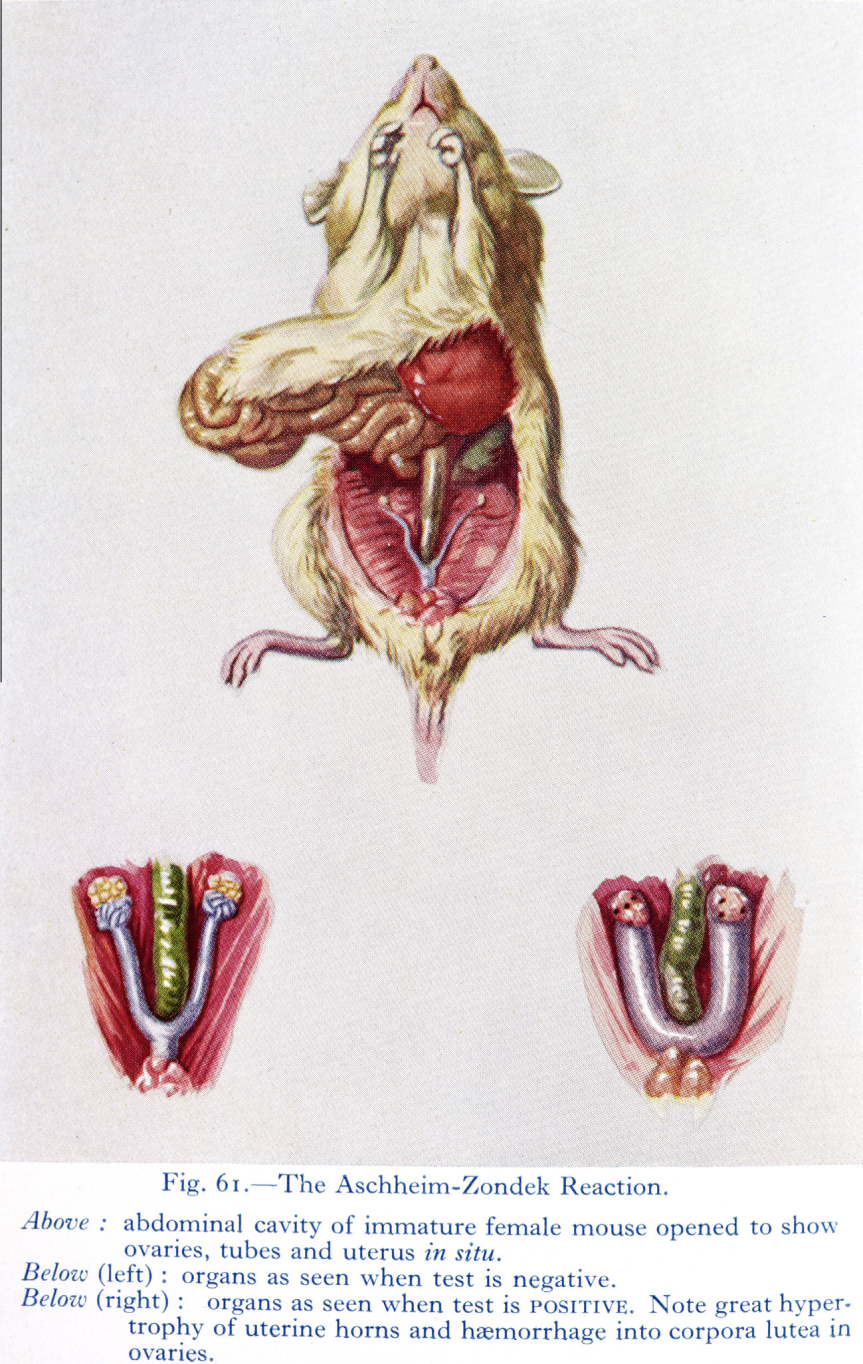
A colourful illustration of the Aschheim–Zondek reaction from the seventh edition of Johnstone’s popular textbook (Johnstone, 1934, unpaginated plate between pp. 82 and 83). Reproduced by kind permission of the Syndics of Cambridge University Library.

The Aschheim–Zondek test, Johnstone later quipped, raised mice to the ‘rank of obstetrical consultants’ (Johnstone, 1947, p. 11). The increasing demand for laboratory mice was met in Britain chiefly by the specialist commercial breeder and distributor, A. Tuck & Son’s ‘Mousery’ in Rayleigh, Essex (Kirk, 2008, p. 285). The agricultural correspondent of the *News Chronicle* called Mr Tuck ‘the uncrowned king of mice fanciers’ and the *Daily Mirror* reported that his ‘farm’ housed 200,000 mice and dispatched up to 3,000 ‘of all sizes, shapes and colours’ daily (quoted in Bayly, 1936, p. 25). Tuck supplied young, female mice for use in Edinburgh, where Crew’s staff initially followed Aschheim and Zondek’s original technique to the letter. They first injected a batch of five mice with urine extract twice a day for three days in a row (a total of thirty injections). Next they sacrificed and dissected each animal to visually inspect their reproductive systems. Laboratory workers interpreted the presence of sexually mature organs (especially ovarian ‘blood spots’) in at least one mouse as a positive reaction. Immature organs meant a negative result (Fig. 2). Aschheim and Zondek intended the use of multiple test animals to mitigate the variability of individual mice and so increase the sensitivity of their test, which required several days to perform because infant mice would not tolerate an injection of the required amount of extract all at once. Preparing the urine was also time consuming, but failing to do so often resulted in dead mice before a conclusive result could be obtained.

Crew’s staff initially sectioned the ovaries and inspected them under a microscope. To further simplify, streamline, and speed up the procedure, they soon abandoned microscopy in favour of naked-eye inspection, which was usually adequate. In borderline cases, an intact ovary could be pressed between cover-slips and examined under a hand-lens or held up to the light, where small and deeply embedded blood points could usually be distinguished from even the densest yellow bodies without going to the trouble of slicing (Crew, 1930). For the first three months, Crew and Wiesner tested urine specimens provided by Johnstone and then, satisfied with their results, they decided to go postal.

## 3 Going postal and redescribing errors

In October 1928, a *Lancet* editorial first mentioned the Aschheim–Zondek reaction as a ‘specific’ new test for the ‘presence or absence’ of early pregnancy. The editorial anticipated the ‘very great value’ of the test, assuming the promising results obtained in Berlin would be ‘confirmed by other workers.’^18^ A few months later the *Lancet* and the *British Medical Journal* (*BMJ*) carried a letter from Johnstone explaining that by indiscriminately testing any specimen sent by a doctor, Crew and Wiesner would investigate the sensitivity and specificity of the Aschheim–Zondek test. This was said to be trustworthy from two weeks after a missed period and the only requirements were a few ounces of urine, a covering letter with clinical data, and a postal order for the fee. Results would be returned in about a week (Johnstone, 1929a, 1929b). A supportive *BMJ* editorial amplified Johnstone’s hope that many doctors would take advantage of the station and endorsed the fees as ‘very moderate’. Laboratories in Germany and other countries were beginning to test the test and to publish their reports in research journals (Table 1). However, the editorial argued that a large-scale trial on unselected material was still needed to confirm the ‘clinical value’ of the test in Britain.^19^

In the six weeks following the publication of Johnstone’s letters, the station received around ninety specimens. This was a fair start but there were some logistical problems, so Crew provided additional guidelines in another letter. Mice had to be purchased and looked after and some doctors failed to pay up, so he reminded them that the service was not free. Private cases were charged a ‘modest fee’ of five shillings, intended to permit a reduced hospital fee of one and six.^20^ The station required two ounces of fresh morning urine in a clean bottle enclosed in a sturdy package, accompanied by case notes, especially the date of the patient’s last menstrual period, but doctors frequently posted ‘too much, too little, or too stale urine,’ often in packages that broke in transit (Crew, 1929a, 1929b).

The General Post Office, Britain’s largest employer in the 1920s, allowed urine and other normally prohibited substances to be sent to any recognised medical institute or qualified practitioner (Griffiths, 1997, p. 678). Diagnostic laboratories typically appointed a medical superintendent to oversee operations, a position filled by Edwin Robertson in Edinburgh. Every year, tens of thousands of packets containing pathological specimens (mostly urine) circulated in the post. Many reached the Clinical Research Association (CRA), a large London-based commercial laboratory that supplied doctors with regulation containers and ready-addressed envelopes or boxes for return (Worboys, 2004). The frequency of broken and spilled packages induced the Postmaster General repeatedly to specify regulations in the *BMJ* (Cunningham, 1992, p. 311). Specimens needed to be securely packed in a strong wooden, leather, or metal case to prevent shifting about and with sufficient absorbent sawdust or cotton wool to prevent leakage. The container had to be conspicuously marked ‘Pathological specimen—Fragile with care’, and any packet found to contravene regulations would be destroyed and the sender liable to prosecution.^21^

Nurtured by the requirements of life assurance companies for urinalysis, the CRA and other commercial labs scaled-up diagnostic services to meet an increasing demand from doctors (Dupree, 1997, p. 100; Worboys, 2004). Pregnancy diagnosis cost about as much as haemoglobin estimation or Wassermann’s reaction, which ranged from two shillings a test for panel patients and their dependants to ten and six for the well-heeled (Foster, 1983, pp. 32–37). Specimens that survived the trip to Edinburgh were filtered on arrival by laboratory workers into numbered bottles. Crew’s staff then entered the particulars in a special logbook with perforated pages to produce numbered labels for the urine container and mouse cage, record cards for injection and filing, and ‘result’ and ‘follow-up’ letters. No later than six days after receipt of specimen, a secretary would post the ‘result’ letter to the sender. Two months after that, she would post a reminder letter to find out if the doctor had corroborated or contradicted the laboratory diagnosis by clinical evidence of pregnancy or its absence.^22^

Other labs had reported a disturbingly large error of up to 5%, which provoked debate over the specificity and clinical value of the Aschheim–Zondek reaction. Delegates from the Edinburgh station defended the test in January 1930 at a London meeting of the prestigious Royal Society of Medicine. John Hannan, a registrar at the Soho Hospital for Women had used rats instead of mice and reported a 7% error. He doubted the usefulness of any test that was not ‘absolutely reliable’ and preferred the ‘old method of seeing the patient in a month’s time’ (Hannan, 1930, p. 637). Wiesner insisted that the Aschheim–Zondek reaction could only be evaluated fairly if the original unmodified method was tested with ‘sufficient material collected under clinical conditions.’ This had been done, he claimed, not in London, but in Edinburgh, where the error was a satisfactory 2%. But he emphasised that a positive result was ‘a sign of placental activity’ only and looked forward to the day when a ‘chemical test’ would be able to detect ‘the presence of a living fœtus’. Meanwhile, Wiesner was the first to admit that the Aschheim–Zondek reaction was simply ‘not a pregnancy test, *sensu strict[o]*’ (Hannan, 1930, p. 638).

The influential obstetric surgeon Louis C. Rivett claimed that clinical diagnosis was ‘easy’ in 99% of cases and that an expert could usually handle the doubtful 1% without recourse to the lab. He had provided biochemist Frank Dickens at the Courtauld Institute with over 200 specimens collected from Queen Mary’s Hospital, where East End women competed for limited beds by applying for accommodation at the first sign of pregnancy (Allan & Dickens, 1930). Dickens was reasonably satisfied with the reliability of the test, but like Hannan he discontinued routine testing to free up laboratory animals for more prestigious pituitary research.^23^ Arthur Giles, a well-known gynaecologist at the Chelsea Hospital for Women, amplified Rivett’s criticism about lack of specificity. The test gave positive results for non-pregnant women in a ‘considerable variety of conditions’ and most gynaecologists, he claimed, would probably agree that ‘for the present they had better trust to their fingers and their senses generally for the diagnosis of pregnancy’.^24^ He did, however, praise the ability of the test to detect placental cancer.^25^

Rarely, in the early stages of pregnancy, the fingerlike protrusions of the placental membrane (chorionic villi) transform into bunches of grape-like cysts. As the ‘hydatidiform mole’ grows, the embryo usually dies and is reabsorbed. At first a ‘molar pregnancy’ looks and feels normal, but then the uterus begins to grow abnormally fast and becomes soft and boggy to touch, with no fetal parts to feel, or heartbeat to hear.^26^ Before the Aschheim–Zondek test, the only foolproof diagnostic criterion was a discharge containing tiny cysts, resembling ‘white currants in red currant juice’ (Johnstone, 1934, p. 267). Once diagnosed, a mole could be manually squeezed out, but any retained bits were liable to develop into a highly malignant trophoblastic cancer known as ‘chorionepithelioma’ or ‘chorioncarcinoma’, which could rapidly and fatally spread to the lungs. So following surgical removal or spontaneous delivery, a patient would be instructed to check in regularly for up to a year, or at once if there were any irregular bleeding.

Aschheim had been one of the first to report a positive reaction in a case of chorionepithelioma following the expulsion of a hydatidiform mole (Aschheim, 1929). And, although chorionepithelioma was rare, cancer specialists nevertheless embraced his test as a significant breakthrough in diagnostics.^27^ Early detection and treatment (with some combination of surgery, radium and chemotherapy) was a cornerstone of the early twentieth-century ‘crusade’ against cancer in Britain (Austoker, 1988; Cantor, 2008; Löwy, 2010; Medina-Domenech & Castañeda, 2007; Moscucci, 2009). Yet few general practitioners saw many patients suffering from malignancy, which made early diagnosis a real challenge (Donaldson, Cade, Harmer, Ward, & Edwards, 1936). Hopeful researchers announced new serological tests for cancer on a regular basis and by 1930 over twenty serodiagnostic methods had been proposed (Wright & Wolf, 1930). ‘Unfortunately,’ as Liverpool gynaecologist William Blair-Bell lamented, ‘none had proved specific for malignancy.’ Even as he ‘doubted’ whether ‘science’ would ever produce ‘a test so delicate as to indicate the existence of a few cancer cells in the human body’, he implored ‘biochemical investigators’ to ‘not lose sight of the immense importance’ that would attach to such a discovery.^28^

Robertson, who had also been at the London meeting, echoed Rivett’s hopes for cancer monitoring and control in an address to the Edinburgh Obstetrical Society. One local patient with chest symptoms caused by a metastatic mass had tested positive, demonstrating how repeated testing at regular intervals could be used to monitor the results of surgery or other treatment. Leading Edinburgh gynaecologists were easily persuaded of its value: Theodore Haultain was having one of his patients tested on a weekly basis after she had delivered a hydatidiform mole and James Young proposed that interval testing should be made routine in all such cases. The president of the society congratulated Robertson, who ‘had only to ask’ if he needed specimens, ‘for those who had listened to him and to his facts would be only too glad to help to further the uses of such a test’ (Robertson, 1930, p. 131).

Despite this locally warm reception, however, the Edinburgh station incurred a deficit of £135 in its first year and was threatened with closure. Some doctors had failed to pay up and dozens of tests had been repeated when batches of mice were killed by toxic urine or the visible changes in their bodies were ambiguous. Retesting with second and third specimens was costly and usually fruitless. An increasing demand suggested that the station was ‘appreciated by hospitals and practitioners’, but this did not necessarily justify its continued existence. Wiesner informed the MRC that the station had met its stated research goal of testing the test and that he would need to propose new research aims to justify any continued funding. On the other hand, standards of animal stock had been established and the necessary infrastructure built up to support a routine service independent of any research agenda. This relatively well equipped and smoothly running laboratory was now ‘ready for use by anybody’ willing to uphold the necessary standards.^29^

**Fig. 3:**
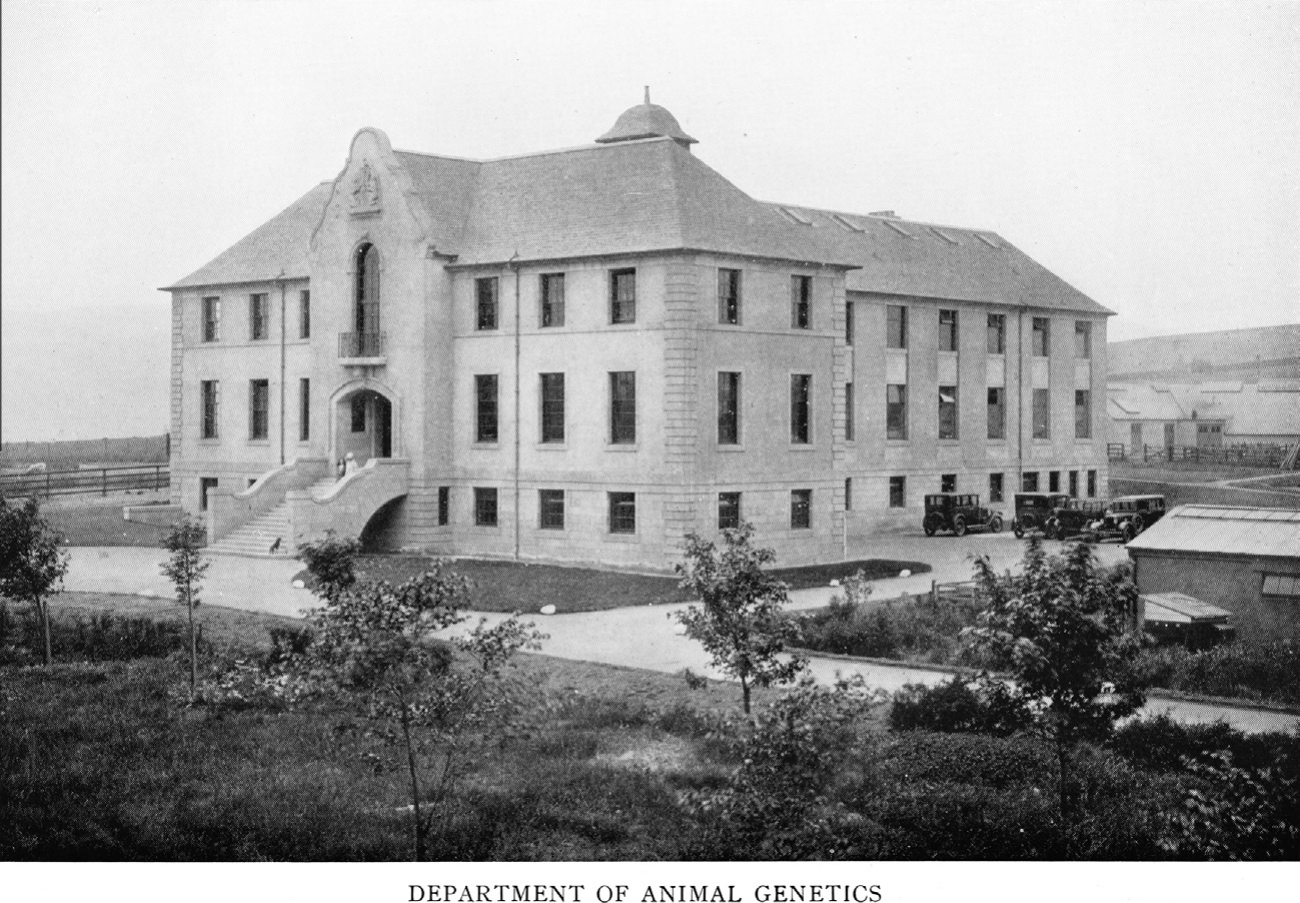
An official photograph of Crew’s institute at the King’s Buildings Site, viewed from the northeast (‘The Department of Animal Genetics’, *University of Edinburgh Journal*, Autumn 1930, 35–40, unpaginated plate between pp. 36 and 37). Reproduced by kind permission of the Syndics of Cambridge University Library.

At this critical juncture, Wiesner was the first to declare that the station could simply be shut down. But he stood by the value of the service and advised its relocation to some other adequately equipped institution, such as the Laboratory of the Royal College of Physicians of Edinburgh. Alternatively, he estimated that doubling the fees would cover expenses in a second year of operation. He also expressed an interest in continuing to work with the test and with the surplus urine it brought him. Crew’s weak position within the British medical establishment, in an agricultural department far from the great London teaching hospitals, enhanced for him the value of Wiesner’s initiative and in the end the station remained in Crew’s institute, which moved into a new building in March 1930 (Fig. 3). Wiesner promised to tighten up his bookkeeping and the MRC agreed to cover the station for a loss of up to £50 for one year only.

Crew’s first annual report announced that fees would be increasing to ten shillings for private cases and three for hospitals (still well within the range of a Wassermann test). This was a winning strategy and in one year the station had become financially ‘self-supporting’, even generating ‘a small balance’ to be ‘carried forward as reserve.’^30^ Crew’s report further clarified the potentially misleading use of the word ‘pregnancy’ in communications by the station. A few doctors had complained that a negative result was followed by miscarriage, proving that the patient had been pregnant (with a dead fetus) at the time of the test (Johnstone, 1930, p. 175). Rather than admit error, Crew creatively reinterpreted ‘false’ negatives as positive indications of a hormonally deficient pregnancy that would probably not go to term (Crew, 1930, p. 662). Far from discouraging, such ‘errors’ opened a window of opportunity for Crew and Wiesner, who began to calibrate the test so that laboratory results would match clinical expectations.^31^ In addition to the asset of ‘false positives’ in cancer diagnosis, they redescribed ‘false negatives’ as positive predictors of ‘fetal death’ and began to remake the Aschheim–Zondek test into a detector of women who were likely to miscarry.

## 4 Clinical pathologists, family doctors, and rabbits

In the late 1920s, the well-connected physician Sir Thomas Horder lamented ‘the existence of laboratories in which the personal element as between doctor and pathologist is quite eliminated’, even as he admitted that they were ‘necessary’ and had ‘come to stay’ (quoted in Lawrence, 1998, p. 99). For best results the *Practitioner* generally recommended working with a local pathologist, rather than relying on a ‘remote laboratory’ (Dukes, 1936), a practice later derided as ‘postal pathology’. Despite the distance, its many southern clients generally welcomed the Aschheim-Zondek reaction and the Edinburgh station. This was a significant achievement at a time when some diagnostic tests were renowned for their ‘great reliability’ and others ‘definitely black-listed.’^32^ The procedure for collecting a specimen was lauded as the ‘simplest imaginable’ (it did not require a catheter as with urine for bacteriological tests) and the manageable error was ‘easily guarded against by ordinary clinical observation.’^33^ One article in the *Clinical Journal* recommended London hospitals for pregnancy testing (Green-Armytage, 1934), but Crew’s service was usually singled out.^34^ Although Liverpool gynaecologist Arthur Gemmell cautioned that the station was not ‘always accurate’ (he had received two incorrect results), he did not reject the test, but instead recalled that it ‘was not a test for pregnancy, but for the presence of living chorion, and that its reported result must be carefully considered in connexion with the clinical findings.’^35^

As we have seen, a few elite gynaecologists trusted their own senses more than a test that gave the wrong answer in one out of every fifty or even twenty cases. But there was no consensus on the error, which varied by laboratory, and Crew and Wiesner were creatively redefining mistakes to convert the liability of non-specificity into the advantage of versatility. Furthermore, family doctors had their own reasons for preferring a postal service to the delicacies of pelvic examination. A note in the *Lancet* in 1930 recommended the Aschheim–Zondek test as ‘sufficiently reliable for all clinical purposes,’ and for ‘the further advantage that in delicate circumstances it can be done without the knowledge of the patient or her friends.’ The note predicted that, although the ‘technique needs practice’, it was ‘likely to be acquired by clinical pathologists’ now that its ‘value’ had been ‘confirmed’. ‘The family doctor’, it concluded, ‘will be grateful for the simplicity of his share, which consists only in collecting morning urine from the patient and possibly adding a drop of tricresol as a preservative.’^36^

For the ordinary family doctor, pelvic examination was complicated by the ever-present possibility of normal pregnancy, which generally needed to be confirmed or excluded. The classical signs included amenorrhea, nausea, sore breasts and ‘quickening’, when a mother begins to feel the fetus move sometime in the second trimester (Duden, 1992). Light bleeding, however, could complicate a diagnosis and the presence of fibroids challenged even ‘the most erudite’ (Green-Armytage, 1934, p. 53). The most important clinical method of early pregnancy diagnosis was the ‘bimanual’ technique of eliciting ‘Hegar’s sign’, a soft, compressible area between the cervix and the uterus (Oakley, 1984, p. 25). But internal examination at an early stage risked inducing miscarriage and, perhaps more importantly, a mutual feeling of ‘delicacy and sensitiveness’ between a patient and her doctor strongly discouraged the practice of pelvic examination unless absolutely necessary.^37^

Prior to the Aschheim–Zondek test, the most promising alternative to the intimacies of physical examination was radiography. A pioneering American handbook on obstetric radiography praised X-rays as ‘a very valuable aid in the diagnosis of pregnancy’, especially for differential diagnosis, but also to ‘dissipate’ the ‘scandalous’ stories told by ‘venomous gossip-mongers’ about ‘single women or widows,’ as well as in court, for settling law-suits, libel cases, and ‘to disprove charges made in actions for divorce’ (Dorland & Hubeny, 1926, p. 259; Howell, 1995, pp. 149–150; Oakley, 1984, p. 100). Fetal bones, however, did not cast shadows until about the sixteenth week of gestation and the demand for X-rays in pregnancy diagnosis significantly declined following the introduction of pregnancy testing.^38^ Later in pregnancy and so also of little use for early diagnosis, the outline and movements of the fetus could be felt by palpation and the fetal heartbeat heard by auscultation (Herschkorn-Barnu, 2002).

In the early 1920s, the fourth edition of Johnstone’s popular *Text-book of Midwifery* briefly mentioned that Abderhalden’s serum test for pregnancy was ‘of theoretical interest only’, because its ‘difficulty’ made it ‘impracticable in all but exceptional cases’ (Johnstone, 1923, p. 93). A decade later, most standard textbooks provided practical instructions on how to collect and post a urine specimen for pregnancy diagnosis. For instance, the second edition of Haultain and Fahmy’s *Ante-natal care* claimed that the Aschheim–Zondek test could be performed only ‘in a laboratory, by expert observers’, and specifically mentioned Edinburgh (Haultain & Fahmy, 1931, p. 31). The sixth edition of Johnstone’s textbook instructed doctors to post specimens, a brief history, and ten shillings to the ‘Pregnancy Diagnosis Station, University—King’s Buildings, Edinburgh’ (Johnstone, 1932, p. 83). The fourth edition of Blair-Bell’s *Principles of gynaecology* enthusiastically proclaimed that the Aschheim–Zondek test had ‘revolutionized’ pregnancy diagnosis (Blair-Bell, 1934, p. 149). Aleck Bourne’s *Midwifery for nurses*, recommended as a study guide for the Central Midwives Board examination, suggested posting urine to a given address or to Edinburgh ‘with the name and age of the woman, the date of dispatch, date of her last menstruation, and a postal order for 10*s*’ (Bourne, 1935, pp. 68–69).

As with X-rays and the Wassermann test in mass screening, the cost of an Aschheim–Zondek test decreased as demand increased (Davis, 2008; Macleod, 1936). But some critics objected to the organisation of pregnancy testing in Britain. In his public speech at the opening of Crew’s institute in 1930, Sharpey-Schafer complained that the resources of a research institute ‘should not be diverted to a routine method of diagnosis which might as well be done anywhere else’ (Sharpey-Schafer, 1930, p. 31), a complaint that was repeated in the *Scotsman* under the subheading ‘Certificate for a mouse.’^39^ Crew’s institute was licensed for vivisection, but pregnancy testing as such was not specifically addressed by the Home Office until 1932, when an inspector advised a doctor to obtain a license and Certificate A, setting a precedent for subsequent would-be pregnancy testers.^40^

On the other hand, even as the *BMJ* complained that doctors were forced to rely on ‘special centres’ that concentrated and maintained ‘large stocks of mice’ and ‘skilled service’, it doubted that pregnancy testing would ever become practical as a side-room technique. So the search continued for the ‘ideal test’, one that was not ‘unpleasant to patient or physician, but simple, capable of being used by the geographically isolated general practitioner, cheap in time and money, and, of course, reliable.’^41^ Researchers at London hospitals and Crew’s student Cecil Voge in Edinburgh investigated cheap, quick, and simple biochemical reactions, but after hundreds of tests on surplus pregnancy urine they were forced to admit that infant mice beat their *in vitro* tests (Hannan, 1930; Voge, 1929). Others experimented with adult mice and (male and female) rats, but the next major breakthrough came in 1931 when researchers in Philadelphia announced a new rabbit test (Friedman & Lapham, 1931).

The ‘Friedman test’ used one or two large, female adult rabbits instead of a batch of five tiny, immature mice. Because rabbits only ovulate immediately after mating (or when one doe ‘jumps’ another), an isolated animal with a known history could be used at any time without fear of a false positive from spontaneous ovulation. Rabbits, like mice, had to be sacrificed, but were comparatively easy to handle and inject in the ear-vein, an already standard procedure in bacteriological testing and vaccine production. They could also tolerate larger doses of urine and soon became the pregnancy-test animal of choice in American laboratories (Leavitt, 2006). Compared to mice, housing rabbits individually in cages (to prevent ovulation) was expensive and required more space, but Friedman’s test dramatically reduced the waiting time for a result from five days to twenty-four hours, offering doctors a more flexible service in urgent cases.

The Edinburgh station soon experimented with the Friedman test, charging one pound, ten shillings to private doctors and one pound to hospitals (around fifty and thirty-three pounds respectively in 2005 money) to cover the higher cost of rabbits and telegraphic communication of the results (Wiesner, 1931, 1932). Contrary to Crew’s expectations, demand for Friedman testing in Edinburgh remained low, mainly because it was too expensive and because large teaching hospitals in London and other cities managed to establish facilities of their own (Table 2).^42^ Crucially, the use of rabbits facilitated the establishment of local alternatives to Crew’s remote (for clients outside Scotland) service. Peter Bishop, a clinical endocrinologist at Guy’s, modified the Friedman test by introducing a delicate surgical procedure to identify spontaneous ovarian blood spots that might otherwise have led to a misdiagnosis (Bishop, 1932, 1933, 1934). This involved operating on each rabbit before and after every test.

**Fig. 4:**
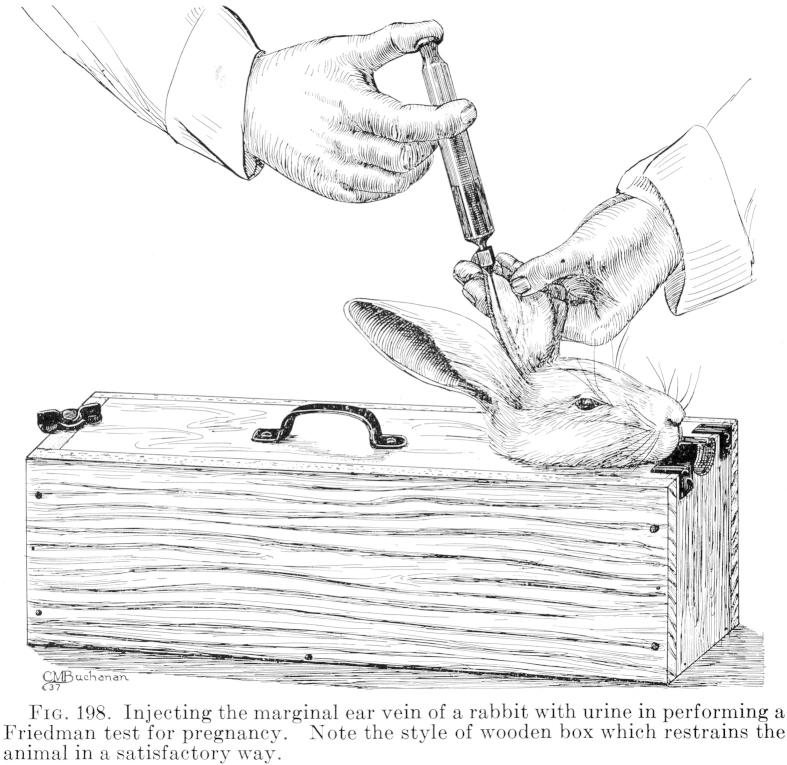
Line drawing of a rabbit injection with restraining box in Roy Kracke’s *Textbook of clinical pathology* (Kracke, 1938, p. 513). Reproduced by kind permission of the Syndics of Cambridge University Library.

By 1935 most London teaching hospitals were equipped for the Friedman test. Ronald Kelson Ford’s *Short ante-natal and post-natal handbook* called it the ‘more generally used’ pregnancy test in Britain (Ford, 1935, p. 6), and the *BMJ* claimed it was ‘well established in clinical midwifery practices.’^43^ A pathologist at St. Thomas’s Hospital praised the ‘much simpler’ Friedman test, reporting over 700 reactions in 1936 (Bamforth, 1936, p. 132). Unlike ‘delicate to handle’ and ‘difficult to obtain’ mice, rabbits were ‘much more satisfactory’ to work with at St. John’s Hospital, Lewisham. There, a specially constructed box was used to bunch up the rabbit’s back and prevent it from kicking at one end while holding its neck between two boards ‘after the manner of an old-fashioned pillory’ at the other (Ralph, 1934, p. 57) (Fig. 4). Bishop’s modified technique was considered impractical in Edinburgh, where Friedman’s test was combined with a confirmatory Aschheim–Zondek test, a control that required ‘much less surgical skill’ (Crew, 1936a, p. 993). The Edinburgh station had been made for mice, which were more convenient to house on a large scale. Rabbits, in contrast, were locally expensive, ‘difficult to breed, to procure, and to accumulate in large numbers’ (Crew, 1937, p. 990). In Crew’s words, different tests were ‘equally satisfactory in the hands of different people’ (Crew, 1936b, p. 1093). When it came to pregnancy testing (and probably diagnostic tests more generally), each lab implemented its own protocols, locally adapted to suit particular needs and constraints.

## 5 Calibrating mice for diagnostic versatility

Even as Johnstone claimed that the station was ‘not a commercial undertaking,’ and that it served ‘the interest of the [medical] profession and of science’ (Johnstone, 1933, p. 557), Wiesner’s research programme had become marginalised within Crew’s institute and was finally shut down in 1934. Crew had come under increasing government pressure to use his national funds for work with farm animals only and the economic depression dried up Macaulay’s money (Deacon, 1979). The new financial situation strained Crew’s relationship with Wiesner, whose work on sex hormones had embarrassingly led to the development of a placenta-based drug by their chief competitor, the Montreal biochemist James B. Collip (Li, 2003). Crew later recalled that Wiesner’s research on the maternal behaviour of rats (Wiesner & Sheard, 1933), which had little relevance to ‘either animal genetics or animal breeding’, was ‘getting out of hand’ and so Crew was not ‘unhappy to see it come to an end.’^44^

Wiesner moved to London to set up an infertility clinic with his second wife Mary Barton (Lane-Roberts, Sharman, Walker, & Wiesner, 1939; Pfeffer, 1987, 1993). Artificial insemination by donor was becoming more widely used in British clinics as a medical fix for male infertility in married couples and Wiesner integrated the Aschheim–Zondek reaction (as an early pregnancy test) into infertility diagnosis and treatment regimes.^45^ He also circularised clients of the Edinburgh station to inform them that he was taking it with him to London. Crew responded in the *BMJ* that testing would not stop just because Wiesner was leaving. The station was larger than ‘the personal activities of one man’, and would continue under the supervision of Wiesner’s assistant, John M. Robson. Though centrally located by Scottish standards, Crew’s station was financially dependent on custom from London and the South of England. Scaling up had made the service financially viable, but also vulnerable to competition as thousands of tests had to be made annually to cover the running costs. To keep serving Scotland, Crew would have to serve England as well and he was unwilling to give up that lucrative share of his market without a fight. Crew admitted that if endocrinology were a more advanced science ‘there would of course be room for more diagnostic laboratories’. But for now, he claimed, a centralised, non-commercial service was needed to produce knowledge about the ‘unusual’ and ‘exceptional’ cases that would someday lead to new breakthroughs in hormone therapy (Crew, 1934, p. 531).

By 1936, the Aschheim–Zondek test was ‘becoming one of the everyday tools of the practitioner’.^46^ The third edition of *Recent advances in endocrinology* called it ‘probably the most accurate biological test known’ (Cameron, 1936, p. 331). A handbook for general practitioners on the early diagnosis of cancer claimed it was ‘so reliable that a positive result must be accepted as proof of the presence of chorion epithelioma’ (Donaldson et al., 1936, pp. 28–29). Even the previously sceptical Hannan had begun to recommend fortnightly interval testing in ‘all cases where the histological picture is suggestive of chorion carcinoma’ (Hannan, 1933, p. 1047). Crew declared that the ‘widespread demand’ for pregnancy diagnosis had been ‘successfully met’ and predicted that ‘as their usefulness [became] more generally known’ the number of tests performed every year would continue to ‘increase’ (Crew, 1936b, p. 1092). Competition had intensified, but so too had demand.

The unique selling point of Crew’s station over competitors was the degree to which laboratory workers calibrated test mice to produce ‘a graded series of reactions ranging from a “strong” positive through the ordinary “standard” to “weak” and “extremely weak” positives, [ … ] to the ordinary unequivocal negative.’ Graded results produced information beyond the ‘existence or non-existence of normal pregnancy’ by showing ‘the difference between an exceptionally low hormone concentration and the “normal” concentration in cases of early pregnancy, [ … ] thus [disclosing] the threat of imminent abortion.’ They could also ‘distinguish between true pregnancy and the endocrine repercussions of abnormal emotional states, and between pregnancy and menopausal conditions’ as well as track the ‘stages of recrudescence of chorion epithelioma and hydatidiform mole.’^47^

In the case of a suspected placental mole or malignancy, the station also offered special dilution tests. For example, an Edinburgh lab report sent to Alan Brews, a leading gynaecologist at the London Hospital, stated: ‘We have examined the specimen of urine and have found that the concentration of gonadotrophic hormone is very high, dilutions of 1 in 200, giving positive reactions when the normal doses are employed. The result supports your diagnosis of chorion-epithelioma’ (Brews, 1935, p. 1225). Others complained that in their hands the test was ‘capricious’, but Brews emphasised its value ‘as an aid to diagnosing [hydatidiform mole] and as a means of excluding the subsequent growth of a chorion-carcinoma.’^48^ By 1939 he had used the Aschheim–Zondek test in six cases, ‘where no part of the mole had escaped from the uterus; in 5 a positive reaction was obtained in a dilution of 1/200 (in 1 case up to 1/800) and in the remaining case a negative reaction was obtained in undiluted urine.’^49^

**Fig. 5:**
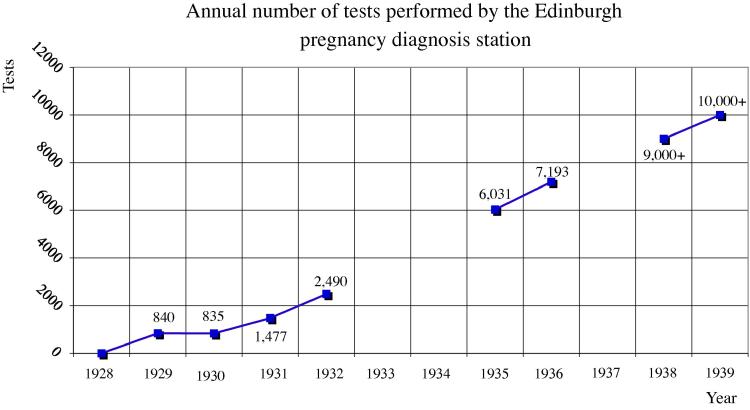
Chart based on published and unpublished annual reports (Crew, 1930, 1936a, 1937; Wiesner, 1931, 1932, 1933). For comparison, the total number of diagnostic tests of all kinds performed by the older Laboratory of the Royal College of Physicians of Edinburgh was 14,798 in 1929 and 16,714 in 1939 (Ritchie, 1953, p. 154).

The number of urine specimens sent to Edinburgh for pregnancy testing increased from around 840 in 1929 to over 10,000 in 1939 (Fig. 5).^50^ About half the demand came from private cases, the other half from hospitals. About half were for non-pregnant women (negative results), many of whom were near menopause. The other half tested positive. Although I have found no records that further break down this demand quantitatively, it is possible to put together a qualitative picture from published reports. Doctors called on the station when patients were unmarried, when obesity or vaginismus impeded ordinary physical examination, in cases of unusual amenorrhoea or vomiting, if fetal death was suspected, and when differential diagnosis was difficult, for instance between ordinary pregnancy and an abdominal tumour, ectopic pregnancy, pseudocyesis (phantom pregnancy), or fibroids. They also requested tests when therapeutic abortion was indicated as by tuberculosis or toxaemia (pre-eclampsia) and, occasionally, in medicolegal circumstances—to establish or exclude pregnancy in cases of criminal abortion, rape, or divorce.^51^ Sometimes a doctor requested a test for allegedly domestic reasons as when a woman was planning to ‘accompany her husband’ to the tropics, but would stay home instead if she happened to be pregnant (Crew, 1936a, p. 993). For those who could afford it, testing was used to calibrate expensive hormone treatment of infertility (Jeffries, 1935).

The Edinburgh station ‘quite commonly’ received brilliant green urine specimens posted by doctors that were lethally toxic to mice, which Crew attributed to ‘single women’ trying to ‘avoid pregnancy’ by chemical means (Crew, 1937, p. 994). By the end of the decade the station received and refused to test five or six urine specimens every week from women ‘who send it in themselves, or chemists, or men.’^52^ These two or three hundred rogue specimens per year suggest that at least a minority of women had learned of the station, despite the evident lack of publicity.^53^ Crew rejected this demand and continued to deal exclusively with the medical profession in order to maintain the respectability of his diagnostic service.^54^ In practice, however, women who knew about the service and could afford to reimburse a sympathetic doctor could order a test for any reason whatsoever: the Edinburgh service was ‘unrestricted’ in this sense and ‘never made a distinction between the medical and social reasons for doing a test’.^55^

Support for pregnancy testing had gathered momentum by the eve of World War II. For instance, the *Report of the inter-departmental committee on abortion* (1939) recommended ‘that the desirability of expanding the existing facilities for carrying out [pregnancy] tests should be fully explored, with a view to making such facilities more generally available, irrespective of income.’^56^ When the British Congress of Obstetrics and Gynaecology convened in Edinburgh in April 1939, Crew boasted that the large volume of urine handled by his laboratory was ‘a measure of the quality of the service that pregnancy diagnosis offers to the clinician, great numbers of whom regarded it as an essential item of their diagnostic equipment’.^57^ With a view towards further expansion, Regina Kapeller-Adler, a refugee biochemist from Vienna who had recently joined Crew’s team, was working on a promising new histidine reaction (Adler-Kastner, 1998), and Crew prepared to replace his mice and rabbits with *Xenopus*, ‘the toad that has not to be slaughtered.’^58^ Demand had increased to the point that Crew confidently recommended the creation of new facilities in London, Leeds, Manchester, Glasgow, Dublin, and Belfast. In addition to providing routine diagnostic services, these laboratories could also actively research new tests for sex hormones. The future was, Crew punned, ‘pregnant with the promise of great discoveries’.^59^

## 6 Diagnostic consumers

Thirty years ago, sociologist Ann Oakley claimed that the Aschheim–Zondek test launched a ‘modern era’ of obstetric knowledge, which asserted its superiority over that of pregnant women themselves. Yet laboratory scientists did not generally promote the test as a means of extending the medical surveillance of pregnant wombs belonging to normal, healthy women. Instead, they often reminded clinicians that the reaction was a test not for the presence of a fetus, but for hormonally active placental tissue. These reminders were not always intended to undermine others’ ability to diagnose ordinary pregnancy, but to promote the clinical usefulness of the diagnostic laboratory. Following Fleck, I have recovered how the Aschheim–Zondek reaction was made into a clinically useful test, not overnight by its eponymous inventors, but incrementally by the collective labour of laboratory workers. I have also attempted to place the diagnostic laboratory ‘more carefully into a wider social canvas’ (Gooday, 2008, p. 786). As I have argued in this article, the reputation of the Aschheim–Zondek test had more to do with differential diagnosis, malignant disease, and infertility treatment, than with ordinary pregnancy. Diagnostic versatility may have threatened to become a ‘major problem with the test’ (Sengoopta, 2006, p. 281), but Crew and Wiesner made it into a major selling point. This is because doctors, not women, were the predominant diagnostic consumers.

Crucially, most women did not need mice or rabbits to tell them they were pregnant and those who turned to a family doctor were generally advised to wait and see. As late as 1962, a handbook on laboratory services for general practitioners counselled that ‘a few weeks’ delay and re-examination will prove the best test of all’ (Lister, 1962, p. 86). As I show elsewhere, the National Heath Service (NHS) covered pregnancy tests for ‘pathological’ cases, but rejected ‘curiosity’ cases. For a fee, the Family Planning Association (FPA) agreed to test any woman regardless of her motivation, but would only communicate the result to her doctor. The use of *Xenopus* by the NHS and FPA made pregnancy testing more socially acceptable in the 1950s, but only in the years leading up to the 1967 Abortion Act, did private commercial labs begin to serve women directly, not as ‘patients’, but as ‘clients’ (Olszynko-Gryn, in preparation). Despite the rise of antenatal care (Oakley, 1984), the state kept pregnancy testing (like contraception and infertility treatment) at arm’s length and was wary of tacitly sanctioning criminal abortion by making an early diagnostic service widely available. From the state’s perspective, a woman could simply wait to find out whether she was pregnant or she could pay out of pocket.^60^

Beyond pregnancy testing, I have begun to explore a lost world of laboratory services. We do not yet have an inclusive enough picture of laboratory life to cover ‘not just the cutting-edge research laboratory, but also the ordinary school laboratory, [as well as] those commissioned for standardized testing and calibration, mobile fieldwork, diagnostic medical analysis, and industrial quality control’ (Gooday, 2008, p. 788). For instance, the literature on cancer is largely silent about serological tests, the most famous of which, ‘Bendien’s test’, caused a sensation in the 1930s (Panton, 1937, p. 793). Historians of postwar biomedicine tend to focus more on biological research than routine services.^61^ And although in the late 1970s the U.S. diagnostic laboratory industry was worth billions of dollars and was roughly the same size as the pharmaceutical industry (Creager, 2008, p. 216), we know comparatively little about it. To better explain the rise of scientific medicine, we need to start recovering the history of diagnostic laboratories—how they were set up and maintained, how they worked in practice, and how the services they offered changed, for example, before and after the creation of the NHS.

The Aschheim–Zondek reaction was made into a routine diagnostic tool in the period when laboratory testing became ‘deeply embedded in medical culture’ (Sturdy, 2011, p. 740). It may have been ‘unwieldy’ for ‘regular use or mass-production’ (Leavitt, 2006, p. 322), yet it was made practical and efficient, streamlined and scaled-up in Edinburgh and elsewhere. In the first half of the twentieth century, new and esoteric practices, including injecting living animals with women’s urine, became the norm in laboratory work. Urine specimens routinely travelled by post. Though less prestigious than scientific research, routine analysis became an essential feature of scientific medicine. As I have argued in this article, demand for the Aschheim–Zondek test was driven less by the medicalisation of pregnancy or the managerial state than by medical entrepreneurs and diagnostic consumers, in this case women and more especially their doctors, who were increasingly willing and able to pay for laboratory services in the 1930s.

## Figures and Tables

**Table 1:** Numbers of pregnancy tests by location of laboratory and in order of descending number of tests; adapted from Zondek (1931, p. 315).

Place	Number of tests
Berlin (Aschheim and Zondek and others)	1200, 200, 109, 12
Frankfurt am Main	1080
Dresden	413
Edinburgh (Crew)	400
Düsseldorf	249
Göttingen	243
London (Dickens)	207
Cologne	139
New York	132, 100
Marburg	129
Breslau	127
Munich	110
Moscow	100
Italy	91, 36, 30
Prague	79, 30
Kiel	51
Münster	49
Greifswald	46
Würzburg	36
Utrecht	33
Vienna	30
Paris	30
Buenos Aires	24
Total	5515

**Table 2:** Numbers of pregnancy tests by kind of test, hospital, and published source, 1930–38.

Number	Test	Hospital	Source
700+	Friedman	Guy’s	Bishop (1934)
700+	Friedman	St Thomas’s	Bamforth (1936)
395	Friedman	Guy’s	Bishop (1933)
380	Biochemical	University College	Dodds (1930)
265	Biochemical	St. Bartholomew’s	Hannan (1930)
237	A–Z	Queen Mary’s	Allan and Dickens (1930)
234	Intradermal	Royal Free	Keevil (1937)
180	Biochemical	University College	Dodds (1936)
147	Intradermal	Middlesex	Gill and Howkins (1937)
98	Friedman	Soho Hospital for Women	Hannan (1930)
65	Biochemical	Charing Cross	Patterson (1937)
53	Friedman	University College	Dodds (1931)
50	A–Z	St. Bartholomew’s	Brewer (1934)
25	Intradermal	General Lying-in (Portsmouth)	Way (1937)
?	Friedman	St John’s	Ralph (1934)


## Footnotes

1 Though later attacked as a fraud, the most famous pregnancy test in the years around World War I was Abderhalden’s serum reaction for ‘protective enzymes’: Abderhalden (1914) and Kaasch (2000). Others included a cobra venom reaction as well as glycosuria, vaginal smear, and skin reaction tests.

2 Henriksen (1941), Bruehl (1952), Johnstone (1954), Cianfrani (1960, pp. 408–409), Hurry (1982), Medvei (1993, pp. 224), O’Dowd & Philipp (2000, pp. 85–86), Burnstein & Braunstein (1995), Shampo (2001), Bröer (2004), Jones & Craft (2004), Wide (2005), Leavitt (2006), Layne (2009), Haarburger & Pillay (2011), Marcus (2011), Tone (2012) and Childerhose & MacDonald (2013). See also the website, ‘A thin blue line: the history of the pregnancy test’, http://history.nih.gov/exhibits/thinblueline/, accessed 5 August 2013.

3 Sengoopta (2006, p. 281). Men with certain testicular tumours also tested positive: Finkelstein & Zondek (1966, p. 9), Leavitt (2006, p. 321) and Han (2013, p. 12).

4 For a discussion of the ultracentrifuge as a versatile tool: Rheinberger (2010, p. 130).

5 On the merits of use-based history of technology, with countless military examples and few from medicine: Edgerton (1999, 2006, 2010).

6 The Pap smear was announced in 1928 and later used in mass screening programmes for cervical cancer. See, for example, Singleton & Michael (1993), Clarke & Casper (1996) and Löwy (2010).

7 For a recent review of the ‘market turn’ in science and technology studies: Simakova (2013, pp. 1–15).

8 Fleck (1979, p. 24). See also Löwy (1993, 2004). On ancient and medieval urine tests for pregnancy: Bayon (1939), Forbes (1957), Oakley (1984, p. 19), Porter & Hall (1995, p. 45), Fissell (2003, 64–65) and Stolberg (2009, 106–116).

9 On Macaulay: Li (2003) and Smith (2009). On the sex congress: Marcuse (1927), Riddle (1927) and Dose (2003).

10 On glandular rejuvenation: Hamilton (1986) and Sengoopta (2006). On the Vivarium: Coen (2006) and Logan (2013).

11 Fletcher to Crew, 8 and 15 May 1928, in ‘Sexual hormones: research by Dr B P Wiesner, Edinburgh University, 1928–32’, National Archives, Kew, London (henceforth NA), FD 1/2816. On Johnstone: Peel (1976, pp. 221–223).

12 Fletcher to Wiesner, 13 March 1929 (NA FD 1/2816). On the MRC’s limited support for sex physiology: Thomson (1975, pp. 42–43) and Austoker & Bryder (1989, pp. 27, 49).

13 This cooperative arrangement fits Steve Sturdy’s assessment of the Edinburgh medical school as a place where lab-clinic collaboration, not conflict, was the norm: Sturdy (2007). Sex physiology, as a marginal and weakly institutionalised field, is also notable for its many instances of interdisciplinary collaborations and material exchanges: Oudshoorn (1994) and Clarke (1998). Revisionist historians are generally beginning to challenge the conflict-oriented narrative of the lab-clinic relationship: Hull (2007), Hammerborg (2011), Sturdy (2011) and Wall (2011).

14 As did Zondek, initially in Schering’s testing laboratory: Gaudillière (2010).

15 The University of Manchester operated a profitable diagnostic laboratory and St. Mary’s Hospital housed a commercial vaccine-producing laboratory: Valier (2002) and Chen (1992). The Dutch firm Organon offered free Aschheim-Zondek tests as propaganda and the American JAX Lab performed tests for area physicians to raise funds for in-house research: Oudshoorn (1994, p. 97) and Rader (2004, p. 171).

16 See Johnstone, Wiesner, & Marshall (1932), and the critical editorial, ‘Sex hormone therapy’, *Lancet*, 3 September 1932, p. 525.

17 Rogers (1937). On the standardisation of therapeutic agents made from living organisms: Gradmann & Simon (2010) and von Schwerin, Stoff, & Wahrig (2013). On standard laboratory animals in Britain: Kirk (2008, 2010).

18 ‘Biochemical diagnosis of pregnancy’, *Lancet*, 20 October 1928, pp. 834–835.

19 ‘Diagnosis of early pregnancy’, *BMJ*, 9 February 1929, p. 259.

20 This is respectively equivalent to the spending worth in 2005 of around £8.30 (the cost of an over-the-counter Clearblue Plus pregnancy test) and around £2.50. All estimates of present-day monetary values in this article were arrived at using the National Archives Currency converter, http://www.nationalarchives.gov.uk/currency/, accessed 5 August 2013.

21 Clinical Research Association (1929, p. 168). Many specimens sent in homemade containers were ‘lost in the post’, in other words, destroyed by the postal authority. In Edinburgh, a small hand press was eventually built to extract the urine from the contents (correspondence and all) of broken packets: Crew (1937, p. 996). For a discussion of packaging and posting issues in the case of radium: Rentetzi (2011).

22 On the technical and secretarial staff of medical research laboratories in mid-twentieth-century Britain: Tansey (2008).

23 Dickens did ‘not undertake private work’ and the *Lancet* redirected inquiries from ‘anxious’ practitioners to Crew’s station: ‘Biological diagnosis of pregnancy’, *Lancet*, 11 January 1930, p. 94.

24 ‘Reports of societies’, *British Medical Journal*, 25 January 1930, pp. 150–153, 151. On the importance of ‘trustworthiness’ to MRC clinical trials in this period: Cox-Maksimov (1997, pp. 70–80).

25 The Aschheim-Zondek reaction was neither the first nor the last pregnancy test to be used to detect cancer. In the 1910s and 1920s, Abderhalden’s reaction doubled as a test for cancer and in the 1970s, American scientists invented a new radioimmunoassay for pregnancy while researching a tumour marker for ‘choriocarcinoma’: Vaitukaitis (2004).

26 On molar pregnancies in early-modern Europe: McClive (2002), McClive & King (2007) and Blanarsch (2009).

27 The incidence at the London Hospital, a large teaching hospital, for example, was on average only three cases of hydatidiform mole every year and one case of chorionepithelioma every two years: Brews (1939, p. 814).

28 Blair-Bell (1930, p. 221). Endocrinology and oncology intersected in the use of hormones to diagnose and treat tumour growth: Blair-Bell argued that normal chorionic tissue was malignant because of its capacity to invade maternal tissue (Cramer, 1930; Peel, 1986, p. 31), and the carcinogenic potential of sex hormones was first debated in the 1930s (Gaudillière, 2006; Johnstone, 1933).

29 Wiesner to Thomson, 8 February 1930 (NA FD 1/2816).

30 Wiesner to MRC, 18 February 1931 (NA FD 1/2816).

31 On calibration as the exertion of control over how the results of a test should be interpreted: Collins (1985) and Pinch (1993).

32 ‘Accuracy of laboratory diagnosis’, *Journal of Clinical Research*, October 1934, pp. 141–142, 141.

33 ‘New and valuable tests’, *Journal of Clinical Research*, April 1933, pp. 63–65, 64.

34 As in this specially commissioned article by the staff of the Macaulay Laboratory: ‘Laboratory tests for pregnancy’, *Journal of Clinical Research*, July 1933, pp. 88–90.

35 ‘Reports of societies’, *British Medical Journal*, 28 February 1931, pp. 351–356, 353.

36 ‘A reliable test for pregnancy’, *Lancet*, 4 January 1930, pp. 36–37.

37 Chisolm (1930). For a discussion of similar issues in Victorian diagnostic practice: Nicolson (2011).

38 Claye (1936), Roberts (1938) and Oakley (1984, p. 98). Maternity hospitals in Britain lacked X-ray departments until the late 1930s: Hiddinga (1995, p. 97). From the early 1950s, X-ray pelvimetry was frequently used in late pregnancy to detect potential difficulties with delivery, but not routinely for early diagnosis: Dry (2006, p. 133). From the late 1950s, ultrasound was occasionally used to diagnose early pregnancy: Nicolson & Fleming (2013, p. 139).

39 ‘Professor’s defence of vivisection’, *The Scotsman*, 1 July, p. 6. American commentators blamed antivivisectionists for the centralisation of pregnancy testing in Britain: Harding (1931) and Rock (1932).

40 M. J. A. Giles, ‘The position of pregnancy tests under the Cruelty to Animal Act, 1876’, 1944, *Vivisection, pregnancy tests: legal option as to the necessity for licence coverage, 1943*–*63* (NA HO 45/25145), ‘Vivisection: Crewe [sic], Dr F A E: pioneer in animal genetics and pregnancy testing techniques’, 1921–1954 (NA 45/24715).

41 ‘Laboratory methods for the diagnosis of pregnancy’, *British Medical Journal*, 24 March 1930, p. 962.

42 On the London hospitals: Rivett (1986).

43 ‘Friedman’s pregnancy test’, *British Medical Journal*, 2 February 1935, p. 211.

44 ‘An interview with Francis Albert Eley Crew (1886–1973), geneticist and professor of animal genetics, University of Edinburgh (8 CDs)’, 1969–71, GB 0237 Science Studies Oral History Project Da 55 SCI 1.

45 Richards (2008, p. 211). Today Wiesner is best known as a notorious sperm donor. See, for example, Tom Kelly, ‘British scientist “fathered 600 children” by donating sperm at his own fertility clinic’, *Daily Mail*, 8 April 2012, http://www.dailymail.co.uk/health/article-2126761/Bertold-Wiesner-British-scientist-fathered-600-children-donating-sperm-fertility-clinic.html, accessed 5 August 2013.

46 ‘The female sex hormones’, *British Medical Journal*, 18 July 1936, p. 126. See also Robson (1934a, 1934b). In the mid 1930s, medical journals filled with ‘enthusiastic reports’ of ‘miraculous’ new pregnancy tests, most famously the bitterling test: Crew (1936a, 1936b) and Weisman (1938).

47 Crew (1939, p. 767).

48 ‘Hydatidiform mole’, *Lancet*, 6 April 1935, p. 823.

49 ‘Hydatidiform mole and chorionepithelioma’, *Lancet*, 4 February 1939, pp. 283–285, 284. See also Brews (1939).

50 This scale is comparable, though on the small side, to diagnostic laboratories that specialised in mass bacteriological, biochemical, and serological testing: Ritchie (1953, pp. 66, 80), Foster (1961, p. 120, 1983, p. 36) and Lawrence (2005, p. 196). For perspective, married women in the 1930s produced 600,000 livebirths each year, though the number of pregnancies (including those ending in miscarriage, abortion or stillbirth) was undoubtedly higher: Szreter (1996, p. 428).

51 For example, in the landmark trial, *R v. Bourne*, gynaecologist Aleck Bourne waited for the (positive) result of a pregnancy test, probably from the Edinburgh station, before performing an abortion on his patient, a young rape victim. See Sir Bernard Spilsbury’s report,‘Respecting the diagnosis of early pregnancy’, 16 July 1938, in ‘Bourne, Dr Aleck W: Abortion’ (NA DPP 2/564). On the trial: Brookes & Roth (1994). On Spilsbury: Burney & Pemberton (2011).

52 ‘Evidence of Professor F. A. E. Crew, Inter-Departmental Committee on Abortion, Tuesday, 21 June 1938’, AC paper 53 (NA MH 71/26), p. 7. See also ‘Memorandum of Professor Crew’, AC Paper 138 (NA MH 71/26).

53 In 1931, the socialist feminist Stella Browne proposed that knowledge of the Aschheim-Zondek test should be made available to women (Hall, 2011, p. 178), but it was not discussed much in newspapers, women’s magazine, or advice manuals until the 1960s (Olszynko-Gryn, in preparation). On the general ban on ‘indirect advertising’ in the non-medical press: Morrice (1994), Nathoo (2009, pp. 36–37).

54 In the 1930s, American birth control clinics used the Aschheim-Zondek test to reject pregnant patients: Hajo (2010, pp. 56, 203).

55 B. M. Hobson, ‘Facilities for pregnancy diagnosis in Britain’, 1966. *Laboratory services: Pregnancy diagnosis services, 1946*–*71* (HH 102/858), National Archives of Scotland.

56 Ministry of Health and Home Office (1939, p. 110). On the committee, before which Crew testified: Brookes (1988, pp. 105–132).

57 ‘The eleventh British Congress of Obstetrics and Gynaecology’, *Journal of Obstetrics and Gynaecology of the British Empire*, June 1939, pp. 582–589, 585.

58 Crew, 1939, p. 768. Female *Xenopus* extrude large, visible eggs when injected with pregnancy urine and so did not need to be sacrificed for dissection in the course of a test: Gurdon & Hopwood (2000) and Olszynko-Gryn (2013).

59 Crew (1939, p. 768).

60 On contraceptive consumers in the 1930s: Tone (1996). On the patient-consumer in Britain: Mold (2010, 2011, 2013).

61 See, for example, the contributions to Quirke & Gaudillière (2008).

## References

[b0005] Abderhalden E. (1914). Defensive ferments of the animal organism against substances out of harmony with the body, the blood-plasma and the cells.

[b0010] Adler-Kastner L. (1998). From *personae non gratae* in Vienna 1938 to respected citizens of Edinburgh: A vignette of my parents, Dr Ernst Adler and Dr Regina Kapeller-Adler. Wiener Klinische Wochenschrift.

[b0015] Allan H., Dickens F. (1930). The Zondek and Aschheim test for pregnancy. Lancet.

[b0020] Amsterdamska O., Hiddinga A., Cooter R., Pickstone J. (2003). The analyzed body. Companion to medicine in the twentieth century.

[b0025] Aschheim S. (1929). The early diagnosis of pregnancy, chorion-epithelioma and hydatidiform mole by the Aschheim–Zondek test. American Journal of Obstetrics and Gynecology.

[b0030] Austoker J. (1988). A history of the Imperial Cancer Research Fund, 1902–1986.

[b0035] Austoker J., Bryder L. (1989). Historical perspectives on the role of the MRC.

[b0040] Bamforth J. (1936). Experiences of the Friedman test for pregnancy. St. Thomas’s Hospital Reports.

[b0045] Bayly M.B. (1936). Cancer: The failure of modern research.

[b0050] Bayon H.P. (1939). Ancient pregnancy tests in light of contemporary knowledge. Proceedings of the Royal Society of Medicine.

[b0055] Bishop P.M.F. (1932). The Zondek-Ascheim reaction and the early diagnosis of pregnancy. Guy’s Hospital Gazette.

[b0060] Bishop P.M.F. (1933). The Friedman test for pregnancy: An analysis of the results of a year’s experience and a suggested modification. Guy’s Hospital Report.

[b0065] Bishop P.M.F. (1934). Pregnancy diagnosis. British Medical Journal.

[b0070] Blair-Bell W. (1930). Some aspects of the cancer problem.

[b0075] Blair-Bell W. (1934). Principles of gynaecology: A textbook for students and practitioners.

[b0080] Blanarsch M. (2009). Die Arzt-Patienten-Beziehung zu Beginn des 18. Jahrhunderts, untersucht anhand Johann Storchs Kasuistik zu Molenschwangerschaften. Medizin, Gesellschaft und Geschichte.

[b0085] Borell M. (1985). Organotherapy and the emergence of reproductive endocrinology. Journal of the History of Biology.

[b0090] Borell M. (1987). Biologists and the promotion of birth control research, 1918–1938. Journal of the History of Biology.

[b0095] Bourne A. (1935). Midwifery for nurses.

[b0100] Brewer H.F. (1934). The Aschheim–Zondek reaction. St. Bartholomew’s Hospital Journal.

[b0105] Brews A. (1935). A follow-up survey of the cases of hydatidiform mole and chorion-epithelioma treated at the London Hospital since 1912. Proceedings of the Royal Society of Medicine.

[b0110] Brews A. (1939). Blair-Bell Memorial Lecture January 27th, 1939. Hydatidiform mole and chorion-epithelioma. Journal of Obstetrics & Gynaecology of the British Empire.

[b0115] Bröer R., Barthels M. (2004). Motes, protective ferments and hormones: Pregnancy testing from antiquity to the present day. Senses, sensors and systems: A journey through the history of laboratory diagnosis.

[b0120] Brookes B.L. (1988). Abortion in England, 1900–1967.

[b0125] Brookes B.L., Roth P., Clark M., Crawford C. (1994). Rex v. Bourne and the medicalization of abortion. Legal medicine in history.

[b0130] Bruehl F.S. (1952). The development of pregnancy tests. American Journal of Nursing.

[b0135] Burney I., Pemberton N. (2011). Bruised witness: Bernard Spilsbury and the performance of early twentieth-century English forensic pathology. Medical History.

[b0140] Burnstein J., Braunstein G.D. (1995). Urine pregnancy tests from antiquity to the present. Early Pregnancy: Biology and Medicine.

[b0145] Cameron A.T. (1936). Recent advances in endocrinology.

[b0150] Cantor D. (2008). Cancer in the twentieth century.

[b0155] Chen W., Cunningham A., Williams P. (1992). The laboratory as business: Sir Almroth Wright’s vaccine programme and the construction of penicillin. The laboratory revolution in medicine.

[b0160] Childerhose J.E., MacDonald M.E. (2013). Health consumption as work: The home pregnancy test as a domesticated health tool. Social Science & Medicine.

[b0165] Chisolm A.E. (1930). Diagnosis in gynaecology. Practitioner.

[b0170] Cianfrani T. (1960). A short history of obstetrics and gynecology.

[b0175] Clarke A.E. (1998). Disciplining reproduction: Modernity, American life sciences, and ‘the problems of sex’.

[b0180] Clarke A.E. (2007). Reflections on the reproductive sciences in agriculture in the UK and US, ca. 1900–2000+. Studies in History and Philosophy of Biological and Biomedical Sciences.

[b0185] Clarke A.E., Casper M.J. (1996). From simple technology to complex arena: Classification of Pap smears, 1917–90. Medical Anthropology Quarterly.

[b0190] Claye A.M. (1936). Haemorrhage in early pregnancy. Clinical Journal.

[b0195] Clinical Research Association (1929). The practitioner’s guide to clinical research: A review of recent advances, followed by alphabetically arranged general sections.

[b0200] Close-Koening, T. (2011). *Betwixt and between: Production and commodification of knowledge in a medical school pathological anatomy laboratory in Strasbourg* (*mid-19th century to* 1939) (Ph.D. thesis). Strasbourg.

[b0205] Coen D.R. (2006). Living precisely in fin-de-siècle Vienna. Journal of the History of Biology.

[b0210] Collins H.M. (1985). Changing order: Replication and induction in scientific practice.

[b0215] Cox-Maksimov, D. (1997). *The making of the clinical trial in Britain, 1910*–*1945: Expertise, the state and the public* (Ph.D. thesis) University of Cambridge.

[b0220] Cramer W., Campaign British Empire Cancer. (1930). The metabolism of the trophoblast. Studies on the diagnosis and nature of cancer.

[b0225] Creager A.N.H., Kroker K., Keelan J., Mazumdar P.M.H. (2008). Molecular surveillance: A history of radioimmunoassays. Crafting immunity: Working histories of clinical immunology.

[b0230] Crenner C., Timmermann C., Anderson J. (2006). Private laboratories and medical expertise in Boston circa 1900. Devices and designs: Medical technologies in historical perspectives.

[b0235] Crew F.A.E. (1929). Biological diagnosis of pregnancy. Lancet.

[b0240] Crew F.A.E. (1929). Diagnosis of early pregnancy. British Medical Journal.

[b0245] Crew F.A.E. (1930). Pregnancy diagnosis station: Report on first year’s working. British Medical Journal.

[b0250] Crew F.A.E. (1934). Pregnancy diagnosis station. British Medical Journal.

[b0255] Crew F.A.E. (1936). Notes from a pregnancy diagnosis laboratory (1935). British Medical Journal.

[b0260] Crew F.A.E. (1936). The endocrines in theory and practice: Laboratory tests for the early diagnosis of pregnancy. British Medical Journal.

[b0265] Crew F.A.E. (1937). Notes from a pregnancy diagnosis laboratory (1936). American Journal of Obstetrics and Gynecology.

[b0270] Crew F.A.E. (1939). Biological pregnancy diagnosis tests: A comparison of the rabbit, the mouse, and the ‘clawed toad’ (*Xenopus laevis*) as the experimental animal. British Medical Journal.

[b0275] Cunningham G.J. (1992). History of British pathology.

[b0280] Davis G. (2008). ‘The cruel madness of love’: Sex syphilis and psychiatry in Scotland, 1880–1930.

[b0285] Deacon M. (1979). The institute of animal genetics Edinburgh: The first twenty years.

[b0290] Digby A., Bosanquet N. (1988). Doctors and patients in an era of national health insurance and private practice, 1913–1938. Economic History Review.

[b0295] Dodds G.H. (1930). Value of the bromine test for diagnosis of pregnancy. British Medical Journal.

[b0300] Dodds G.H. (1931). Rapid laboratory test for pregnancy. British Medical Journal.

[b0305] Dodds G.H. (1936). The Visscher-Bowman test for pregnancy. British Medical Journal.

[b0310] Donaldson M., Cade S., Harmer W.D., Ward R.O., Edwards A.T. (1936). The early diagnosis of malignant disease for the use of general practitioners.

[b0315] Dorland W.A.N., Hubeny M.J. (1926). The X-ray in embryology and obstetrics.

[b0320] Dose R. (2003). The World league for sexual reform: Some possible approaches. Journal of the History of Sexuality.

[b0325] Dry S., Schlich T., Tröhler U. (2006). The population as patient: Alice Stewart and the controversy over low-level radiation in the 1950s. The risks of medical innovation: Risk perception and assessment in historical context.

[b0330] Duden B. (1992). Quick with child: An experience that has lost its status. Technology in Society.

[b0335] Dukes C. (1936). Clinical examination of the urine. Practitioner.

[b0340] Dupree M.W. (1997). Other than healing: Medical practitioners and the business of life assurance during the nineteenth and early twentieth centuries. Social History of Medicine.

[b0345] Edgerton D. (1999). From innovation to use: Ten eclectic theses on the historiography of technology. History and Technology.

[b0350] Edgerton D. (2006). The shock of the old: Technology and global history since 1900.

[b0355] Edgerton D. (2010). Innovation, technology, or history: What is the historiography of technology about?. Technology and Culture.

[b0360] Finkelstein M., Zondek B. (1966). Professor Bernhard Zondek: An interview. Journal of Reproduction and Fertility.

[b0365] Fisher K., Eder F.X., Hall L.A., Hekma G. (1999). ‘Didn’t stop to think, I just didn’t want another one’: The culture of abortion in interwar South Wales. Sexual cultures in Europe: Themes in sexuality.

[b0370] Fissell M.E., Rosenberg C.E. (2003). Making a masterpiece: The Aristotle texts in vernacular medical culture. Right living: An Anglo-American tradition of self-help medicine and hygiene.

[b0375] Fleck L. (1979). Genesis and development of a scientific fact.

[b0380] Forbes T.R. (1957). Early pregnancy and fertility tests. Yale Journal of Biology and Medicine.

[b0385] Ford R.K. (1935). A short ante-natal and post-natal handbook.

[b0390] Foster W.D. (1961). A short history of clinical pathology.

[b0395] Foster W.D. (1983). Pathology as a profession in Great Britain and the early history of the Royal College of Pathologists.

[b0400] Franklin S., Roberts C. (2006). Born and made: An ethnography of preimplantation genetic diagnosis.

[b0405] Friedman M.H., Lapham M.E. (1931). A simple, rapid procedure for the laboratory diagnosis of early pregnancies. American Journal of Obstetrics and Gynecology.

[b0410] Gaudillière J.-P. (2005). Better prepared than synthesized: Adolf Butenandt, Schering Ag and the transformation of sex steroids into drugs (1930–1946). Studies in History and Philosophy of Biological and Biomedical Sciences.

[b0420] Gaudillière J.-P., Schlich T., Tröhler U. (2006). Hormones at risk: Cancer and the medical uses of industrially-produced sex hormones in Germany, 1930–1960. The risks of medical innovation: Risk perception and assessment in historical context.

[b0415] Gaudillière J.-P., Gradmann C., Simon J. (2010). The visible industrialist: Standards and the manufacture of sex hormones. Evaluating and standardizing therapeutic agents, 1890–1950.

[b0425] Gill A.M., Howkins J. (1937). The antuitrin S intradermal pregnancy test. British Medical Journal.

[b0430] Gooday G. (2008). Placing or replacing the laboratory in the history of science?. Isis.

[b0435] Gradmann C., Simon J. (2010). Evaluating and standardizing therapeutic agents, 1890–1950.

[b0440] Green-Armytage V.B. (1934). Obstetrical regrets. Clinical Journal.

[b0445] Griffiths J. (1997). Creating, transmitting, and transforming a corporate culture in a public sector enterprise: The general post office, 1920–1990. Business and Economic History.

[b0450] Gurdon J.B., Hopwood N. (2000). The introduction of *Xenopus laevis* into developmental biology: Of empire, pregnancy testing and ribosomal genes. International Journal of Developmental Biology.

[b0455] Ha N.Q. (2011). The riddle of sex: Biological theories of sexual difference in the early twentieth-century. Journal of the History of Biology.

[b0460] Haarburger D., Pillay T.S. (2011). Historical perspectives in diagnostic clinical pathology: Development of the pregnancy test. Journal of Clinical Pathology.

[b0465] Hajo C.M. (2010). Birth control on main street: Organizing clinics in the United States, 1916–1939.

[b0470] Hall L.A. (2011). The life and times of Stella Browne: Feminist and free spirit.

[b0475] Hamilton D. (1986). The monkey gland affair.

[b0480] Hammerborg M. (2011). The laboratory and the clinic revisited: The introduction of laboratory medicine into the Bergen general hospital, Norway. Social History of Medicine.

[b0485] Han S. (2013). Pregnancy in practice: Expectations and experience in the contemporary US.

[b0490] Hannan J.H. (1930). The detection of the presence of the hormone of the anterior pituitary body in the urine as an aid to the diagnosis of pregnancy. Proceedings of the Royal Society of Medicine.

[b0495] Hannan J.H. (1933). Chorionic carcinoma. British Medical Journal.

[b0500] Hanson C. (2004). A cultural history of pregnancy: Pregnancy, medicine and culture, 1750–2000.

[b0505] Harding T.S. (1931). The degradation of science.

[b0510] Hardy A. (2001). Health and medicine in Britain since 1860.

[b0515] Haultain W.F., Fahmy E.C. (1931). Ante-natal care, including the abnormalities associated with pregnancy and a section on postnatal care.

[b0520] Henriksen E. (1941). Pregnancy tests of the past and present. Western Journal of Surgical Obstetric Gynecology.

[b0525] Herschkorn-Barnu P., Duden B., Schlumbohm J., Veit P. (2002). Wie der Fötus einen klinischen Status erhielt: Bedingungen und Verfahren der Produktion eines medizinischen Fachwissens, Paris 1832–1848. Geschichte des Ungeborenen: Zur Erfahrungs-und Wissenschaftsgeschichte der Schwangerschaft, 17.–20. Jahrhundert.

[b0530] Hiddinga, A. (1995). *Changing normality: Pregnancy and scientific knowledge claims 1920–1950, with special reference to the USA* (Ph.D. thesis). University of Amsterdam.

[b0535] Hinz A., Ebert A., Goetze B., Ebert A., Weitzel H.K. (1994). Der Exodus: Robert Meyer, Selmar Aschheim und Bernhard Zondek. Die Berliner Gesellschaft für Geburtshilfe und Gynäkologie 1844–1994.

[b0540] Hogben L.T. (1974). Francis Albert Eley Crew, 1886–1973. Biographical Memoirs of Fellows of the Royal Society.

[b0545] Howell J.D. (1995). Technology in the hospital: Transforming patient care in the early twentieth century.

[b0550] Hull A. (2007). Teamwork, clinical research, and the development of scientific medicine in interwar Britain: The ‘Glasgow school’ revisited. Bulletin of the History of Medicine.

[b0555] Hurry E. (1982). A laboratorian’s view of pregnancy testing. American Journal of Medical Technology.

[b0560] Hutt F.B. (1931). The work of the Department of Animal Genetics of the University of Edinburgh. Journal of Animal Science.

[b0565] Jeffries L.M. (1935). Treatment of repeated abortion. British Medical Journal.

[b0570] Johnstone R.W. (1923). A text-book of midwifery for students and practitioners.

[b0575] Johnstone R.W. (1929). Biological diagnosis of pregnancy. Lancet.

[b0580] Johnstone R.W. (1929). Diagnosis of early pregnancy. British Medical Journal.

[b0585] Johnstone R.W. (1930). The new physiology of menstruation and its practical implications in obstetrics and gynecology. The Joseph Price Foundation Lecture. American Journal of Obstetrics and Gynecology.

[b0590] Johnstone R.W. (1932). A text-book of midwifery for students and practitioners.

[b0595] Johnstone R.W. (1933). Gynaecological aspects of endocrinology. British Medical Journal.

[b0600] Johnstone R.W. (1934). A text-book of midwifery for students and practitioners.

[b0605] Johnstone R.W. (1947). Ballantyne’s ghost. Transactions of the Edinburgh Obstetrical Society.

[b0610] Johnstone R.W., Munro Kerr J.M., Johnstone R.W., Phillips M.H. (1954). The diagnosis of pregnancy. Historical review of British obstetrics and gynaecology, 1800-1950.

[b0615] Johnstone R.W., Wiesner B.P., Marshall P.G. (1932). The therapeutic application of gonadotropic hormones. Lancet.

[b0620] Jones, E. L. (2007). *Abortion in England, 1861*–*1967* (Ph.D. thesis). Royal Holloway, University of London.

[b0625] Jones G., Craft A. (2004). Corporate venturing: The origins of Unilever’s pregnancy test. Business History.

[b0630] Kaasch M. (2000). Sensation, Irrtum, Betrug?—Emil Abderhalden und die Geschichte der Abweherfermente. Acte Historica Leopoldina.

[b0635] Keevil N.L. (1937). Antuitrin S intradermal pregnancy test. British Medical Journal.

[b0640] Kirk R.G.W. (2008). ‘Wanted—standard guinea pigs’: Standardisation and the experimental animal market in Britain ca. 1919–1947. Studies in History and Philosophy of Biological and Biomedical Sciences.

[b0645] Kirk R.G.W. (2010). A brave new animal for a brave new world: The British Laboratory Animals Bureau and the constitution of international standards of laboratory animal production and use, circa 1947–1968. Isis.

[b0650] Kohler R.E. (2008). Lab history. Isis.

[b0655] Kracke R.R. (1938). Textbook of clinical pathology.

[b0660] Lane-Roberts C.S.L., Sharman A., Walker K., Wiesner B.P. (1939). Sterility and impaired fertility: Pathogenesis, diagnosis and treatment.

[b0665] Lawrence C. (2005). Rockefeller money, the laboratory, and medicine in Edinburgh, 1919–1930: New science in an old country.

[b0670] Lawrence C.J., Lawrence C.J., Weisz G. (1998). Still incommunicable: Clinical holists and medical knowledge in inter-war Britain. Greater than the parts: Holism in biomedicine, 1920–1950.

[b0675] Layne L.L. (2009). The home pregnancy test: A feminist technology?. Women’s Studies Quarterly.

[b0680] Leavitt S.A. (2006). ‘A private little revolution’: The home pregnancy test in American culture. Bulletin of the History of Medicine.

[b0685] Li A. (2003). J. B. Collip and the development of medical research in Canada: Extracts and enterprise.

[b0690] Lister U.M., Thomson W.A.R. (1962). Tests for the early diagnosis of pregnancy. Calling the laboratory.

[b0695] Logan C. (2013).

[b0715] Löwy I., Berridge V., Strong P. (1993). Testing for a sexually transmissible disease, 1907–1970: The history of the Wassermann reaction. AIDS and contemporary history.

[b0700] Löwy I. (2004). ‘A river that is cutting its own bed’: The serology of syphilis between laboratory, society and the law. Studies in History and Philosophy of Biological and Biomedical Sciences.

[b0720] Löwy I., Bonah C., Masutti C., Rasmussen A., Simon J. (2009). Producing pharmaceutical standards at the margins: Chemical contraceptives between the laboratory and the field. Harmonizing drugs: Standards in 20th-century pharmaceutical history.

[b0705] Löwy I. (2010). Preventive strikes: Women, precancer and prophylactic surgery.

[b0710] Löwy I. (2011). ‘Sexual chemistry’ before the pill: Science, industry and chemical contraceptives, 1920–1960. British Journal for the History of Science.

[b0725] Macleod D. (1936). Obstetrics and gynaecology. Practitioner.

[b0730] Marcus P. (2011). The evolution of the urine pregnancy test. The Female Patient.

[b0735] Marcuse M. (1927). Sexualforschung: Verhandlungen des 1. internationalen Kongresses Berlin, 10.-16. Oktober 1926.

[b0740] Marie, J. (2004). *The importance of place: A history of genetics in 1930s Britain* (Ph.D. thesis). University College London.

[b0745] McClive C. (2002). The hidden truths of the belly: The uncertainties of pregnancy in early modern Europe. Social History of Medicine.

[b0750] McClive C., King H., Dasen V. (2007). When is a foetus not a foetus? Diagnosing false conceptions in early modern France. L’embryon humain à travers l’histoire: Images, savoirs et rites.

[b0760] McLaren A. (2012). Sex, robots, trees, and test-tube babies in interwar Britain.

[b0765] Medina-Domenech R., Castañeda C. (2007). Redefining cancer during the interwar period: British medical officers of health, state policy, managerialism, and public health. American Journal of Public Health.

[b0770] Medvei V.C. (1993). The history of clinical endocrinology: A comprehensive account of endocrinology from earliest times to the present day.

[b0775] Ministry of Health and Home Office (1939). Report of the inter-departmental committee on abortion.

[b0780] Mold A. (2010). Patient groups and the construction of the patient-consumer in Britain: An historical overview. Journal of Social Policy.

[b0785] Mold A. (2011). Making the patient-consumer in Margaret Thatcher’s Britain. Historical Journal.

[b0790] Mold A. (2013). Repositioning the patient: Patient organizations, consumerism, and autonomy in Britain during the 1960s and 1970s. Bulletin of the History of Medicine.

[b0755] Morrice A. (1994). ‘The medical pundits’: Doctors and indirect advertising in the lay press 1922–1927. Medical History.

[b0795] Moscucci O. (2009). The British fight against cancer: Publicity and education, 1900–1948. Social History of Medicine.

[b0800] Nathoo A. (2009). Hearts exposed: Transplants and the media in 1960s Britain.

[b0810] Nicolson M., Nuttall A., Mander R. (2011). James Young Simpson and the development of physical diagnosis. James Young Simpson: Lad o pairts.

[b0805] Nicolson M., Fleming J. (2013). Imaging and imagining the fetus: The development of obstetric ultrasound.

[b0815] Oakley A. (1984). The captured womb: A history of the medical care of pregnant women.

[b0820] O’Dowd M.J., Philipp E.E. (2000). The history of obstetrics and gynecology.

[b0825] Olszynko-Gryn J. (2013). When pregnancy tests were toads: The *Xenopus* test in the early NHS. Wellcome History.

[b0830] Olszynko-Gryn, J. (in preparation). *Pregnancy testing in Britain, c.1900-67: Laboratories, animals and demand from doctors, patients and consumers* (Ph.D. thesis). University of Cambridge.

[b0835] Oudshoorn N. (1994). Beyond the natural body: An archeology of sex hormones.

[b0840] Panton P.N. (1937). Cancer tests and treatments. Lancet.

[b0845] Parkes A.S. (1966). The rise of reproductive endocrinology 1926–1940. Proceedings of the Society of Endocrinology.

[b0850] Patterson J. (1937). The chemical diagnosis of early pregnancy: A method based upon the detection of oestriol in the urine. British Medical Journal.

[b0855] Peel J. (1976). The lives of the fellows of the royal college of obstetricians and gynaecologists, 1929–1969.

[b0860] Peel J. (1986). William Blair-Bell: Father and founder.

[b0870] Pfeffer N., Stanworth M. (1987). Artificial insemination, in-vitro fertilization and the stigma of infertility. Reproductive technologies: Gender, motherhood and medicine.

[b0865] Pfeffer N. (1993). The stork and the syringe: A political history of reproductive medicine.

[b0875] Pinch T. (1993). Testing, one, two, three-testing: Towards a sociology of testing. Science, Technology & Human Values.

[b0885] Porter D., Porter D. (1997). The decline of social medicine in Britain in the 1960s. Social medicine and medical sociology in the twentieth century.

[b0880] Porter R., Hall L. (1995). The facts of life: Creation of sexual knowledge in Britain, 1650–1950.

[b0890] Prüll C.-R. (1998). Pathology in the 19th and 20th centuries: The relationship between theory and practice.

[b0895] Prüll C.-R. (2003). Medizin am Toten oder am Lebenden? Pathologie in Berlin und in London, 1900–1945.

[b0900] Quirke V., Gaudillière J.-P. (2008). The era of biomedicine: Science, medicine, and public health in Britain and France after the second world war. Medical History.

[b0905] Rader K. (2004). Making mice: Standardizing animals for American biomedical research, 1900–1955.

[b0910] Ralph R.S. (1934). Hormone tests for pregnancy. Journal of Clinical Research.

[b0915] Rapp R. (1999). Testing women, testing the fetus: The social impact of amniocentesis in America.

[b0920] Rentetzi M. (2011). Packaging radium, selling science: Boxes, bottles and other mundane things in the world of science. Annals of Science.

[b0925] Rheinberger H.-J. (2010). An epistemology of the concrete: Twentieth-century histories of life.

[b0930] Richards M. (2008). Artificial insemination and eugenics: Celibate motherhood, eutelegenesis and germinal choice. Studies in History and Philosophy of Biological and Biomedical Sciences.

[b0935] Richmond M.L., Maienschein J., Laubichler M.D. (2007). The cell as the basis for heredity, development, and evolution: Richard Goldschmidt’s program of physiological genetics. From embryology to evo–devo: A history of developmental evolution.

[b0940] Riddle O. (1927). The accomplishments of the first international congress for sex research. Journal of Social Hygiene.

[b0945] Ritchie J. (1953). History of the Laboratory of Royal College of Physicians of Edinburgh.

[b0950] Rivett G. (1986). The development of the London hospital system 1823–1982.

[b0955] Roberts R.E., Shanks S.C. (1938). Diagnosis of pregnancy. A text-book of X-ray diagnosis by British authors.

[b0960] Robertson E.M. (1930). The practicability of the Aschheim–Zondek test for pregnancy. Edinburgh Medical Journal.

[b0965] Robson J.M. (1934). Pregnancy diagnosis in theory and practice. British Medical Journal.

[b0970] Robson J.M. (1934). Recent advances in sex and reproductive physiology.

[b0975] Rock J. (1932). Progress in obstetrics. New England Journal of Medicine.

[b0980] Rogers L. (1937). The truth about vivisection.

[b0985] Rosenberg C.E. (1990). Looking backward, thinking forward: The roots of hospital crisis. Transactions & Studies of the College of Physicians of Philadelphia.

[b0990] Rothman B.K. (1986). The tentative pregnancy: Prenatal diagnosis and the future of motherhood.

[b0995] Rudloff U., Ludwig H. (2005). Jewish gynecologists in Germany in the first half of the twentieth century. Archives of Gynecology and Obstetrics.

[b1000] Schneck P. (1997). Selmar Aschheim (1878–1965) und Bernhard Zondek (1891–1966): zum Schicksal zweier jüdischer Ärzte und Forscher an der Berliner Charité. Zeitschrift für ärztliche Fortbildung und Qualitätssicherung.

[b1005] Sengoopta C. (2006). The most secret quintessence of life: Sex, glands, and hormones, 1850–1950.

[b1010] Shampo M.A. (2001). Tests, signs, and indications of pregnancy. Journal of Pelvic Surgery.

[b1015] Sharpey-Schafer E. (1930). Research in animal genetics. British Medical Journal.

[b1020] Simakova E. (2013). Marketing technologies: Corporate cultures in technological change.

[b1025] Singleton V., Michael M. (1993). Actor-networks and ambivalence: General practitioners in the UK cervical screening programme. Social Studies of Science.

[b1030] Smith A. (2009). Thomas Bassett Macaulay and the Bahamas: Racism, business and Canadian sub-imperialism. Journal of Imperial and Commonwealth History.

[b1035] Soloway R. (1995). ‘The perfect contraceptive’: Eugenics and birth control research in Britain and America in the interwar years. Journal of Contemporary History.

[b1040] Stevens R. (1966). Medical practice in modern England: The impact of specialization and state medicine.

[b1045] Stolberg M. (2009). Die Harnschau: eine Kultur- und Alltagsgeschichte.

[b1050] Stone J.E. (1932). Hospital organization and management: Including planning and construction.

[b1055] Sturdy S. (2007). Knowing cases: Biomedicine in Edinburgh, 1887–1920. Social Studies of Science.

[b1060] Sturdy S. (2011). Looking for trouble: Medical science and clinical practice in the historiography of modern medicine. Social History of Medicine.

[b1065] Sturdy S., Cooter R. (1998). Science, scientific management, and the transformation of medicine in Britain, c.1870–1950. History of Science.

[b1070] Szreter S. (1996). Fertility, class, and gender in Britain, 1860–1940.

[b1075] Tansey E.M. (1994). Protection against dog distemper and dogs protection bills: The Medical Research Council and anti-vivisectionist protest, 1911–1933. Medical History.

[b1080] Tansey E.M. (2008). Keeping the culture alive: The laboratory technician in mid-twentieth-century British medical research. Notes and Records of the Royal Society.

[b1085] Thomson A.L. (1975). Half a century of medical research.

[b1090] Tone A. (1996). Contraceptive consumers: Gender and the political economy of birth control in the 1930s. Journal of Social History.

[b1095] Tone A. (2012). Medicalizing reproduction: The pill and home pregnancy tests. Journal of Sex Research.

[b1100] Usborne C. (2007). Cultures of abortion in Weimar Germany.

[b1105] Vaitukaitis J.L. (2004). Development of the home pregnancy test. Annals of the New York Academy of Sciences.

[b1110] Valier, H. K. (2002). *The politics of scientific medicine in Manchester, c.1900*–*1960* (Ph.D. thesis). University of Manchester.

[b1115] van den Belt H. (2011). The collective construction of a scientific fact: A re-examination of the early period of the Wassermann reaction (1906–1912). Social Epistemology.

[b1120] Voge C.I.B. (1929). A simple chemical test for pregnancy. British Medical Journal.

[b1125] von Schwerin A., Stoff H., Wahrig B. (2013). Biologics, a history of agents made from living organisms in the twentieth century.

[b1130] Wall R. (2011). Using bacteriology in elite hospital practice: London and Cambridge, 1880–1920. Social History of Medicine.

[b1135] Way S. (1937). Antuitrin S intradermal pregnancy test. British Medical Journal.

[b1140] Weisman A.I. (1938). Recent laboratory tests for the early diagnosis of pregnancy. American Journal of Obstetrics and Gynecology.

[b1145] Wide L. (2005). Inventions leading to the development of the diagnostic test kit industry: From the modern pregnancy test to the sandwich assays. Upsala Journal of Medical Science.

[b1150] Wiesner B.P. (1931). Pregnancy diagnosis station: Report on second year’s working. British Medical Journal.

[b1155] Wiesner B.P. (1932). Pregnancy diagnosis station: Report on third year’s working. British Medical Journal.

[b1160] Wiesner B.P. (1933). Pregnancy diagnosis: Report of forth year’s working. British Medical Journal.

[b1165] Wiesner B.P., Sheard N. (1933). Maternal behaviour in the rat.

[b1170] Wilmot S. (2007). From ‘public service’ to artificial insemination: Animal breeding science and reproductive research in early twentieth-century Britain. Studies in History and Philosophy of Biological and Biomedical Sciences.

[b1175] Worboys, M. (2004). Private clinical laboratories in Britain: The clinical research association, 1894–1914. Unpublished paper presented at the American association for the history of medicine annual conference. Madison, Wisconsin.

[b1180] Wright W.M., Wolf C.G.L. (1930). The serological diagnosis of cancer. Part I. Journal of Cancer Research.

[b1185] Zondek B. (1931). Die Hormone Des Ovariums und des Hypophysenvorderlappens.

